# Recent Advances in the Biomedical Applications of Functionalized Nanogels

**DOI:** 10.3390/pharmaceutics14122832

**Published:** 2022-12-16

**Authors:** Kannan Badri Narayanan, Rakesh Bhaskar, Sung Soo Han

**Affiliations:** 1 School of Chemical Engineering, Yeungnam University, 280 Daehak-Ro, Gyeongsan 38541, Republic of Korea; 2 Research Institute of Cell Culture, Yeungnam University, 280 Daehak-Ro, Gyeongsan 38541, Republic of Korea

**Keywords:** nanogel, polymers, functionalization, stimuli-responsive, cancer, theranostics, targeted delivery

## Abstract

Nanomaterials have been extensively used in several applications in the past few decades related to biomedicine and healthcare. Among them, nanogels (NGs) have emerged as an important nanoplatform with the properties of both hydrogels and nanoparticles for the controlled/sustained delivery of chemo drugs, nucleic acids, or other bioactive molecules for therapeutic or diagnostic purposes. In the recent past, significant research efforts have been invested in synthesizing NGs through various synthetic methodologies such as free radical polymerization, reversible addition-fragmentation chain-transfer method (RAFT) and atom transfer radical polymerization (ATRP), as well as emulsion techniques. With further polymeric functionalizations using activated esters, thiol–ene/yne processes, imines/oximes formation, cycloadditions, nucleophilic addition reactions of isocyanates, ring-opening, and multicomponent reactions were used to obtain functionalized NGs for targeted delivery of drug and other compounds. NGs are particularly intriguing for use in the areas of diagnosis, analytics, and biomedicine due to their nanodimensionality, material characteristics, physiological stability, tunable multi-functionality, and biocompatibility. Numerous NGs with a wide range of functionalities and various external/internal stimuli-responsive modalities have been possible with novel synthetic reliable methodologies. Such continuous development of innovative, intelligent materials with novel characteristics is crucial for nanomedicine for next-generation biomedical applications. This paper reviews the synthesis and various functionalization strategies of NGs with a focus on the recent advances in different biomedical applications of these surface modified/functionalized single-/dual-/multi-responsive NGs, with various active targeting moieties, in the fields of cancer theranostics, immunotherapy, antimicrobial/antiviral, antigen presentation for the vaccine, sensing, wound healing, thrombolysis, tissue engineering, and regenerative medicine.

## 1. Introduction

Modern advances in biomedical nanotechnology have sparked the development of novel materials for theranostic approaches, circumventing the adverse effects of chemotherapeutic drug toxicity. The changes to the atomic and molecular level matter influence the material structures and components and help to enhance the physical, chemical, and biological properties [1]. The next-generation interdisciplinary research between the diverse areas of materials chemistry, nanoscience, biomedicine, and engineering leads to the development of one of the novel technological polymeric nanocarriers with various functionalizations. Among different polymeric nanocarriers such as micelles, polymersomes, nanogels, nanocapsules, and dendrimers, polymeric nanogels (NGs) gain importance because of their unique characteristics of both nanodimensionality and hydrogel nature, along with the property of holding large quantities of hydrophilic compounds. These NGs also resemble biological components in their composition due to their high-water content and organic composition [2]. Natural polymers can provide biocompatibility and structures comparable to that of biological tissues by mediating cellular responses, whereas using synthetic polymers can provide mechanical properties and help to modulate the degradability of the nanosystems. The smart use of both natural and synthetic polymers allows nanosystems to overcome their drawbacks and recreate properties of biological material in close approximation for in vivo biological applications [3]. However, the great importance of the NGs lies mainly in their polymeric properties and composition. Moreover, the high degree of biocompatibility, biodegradability, and toxicity of degraded products plays an important role in exerting their characteristics.

Finally, to obtain stable NGs, the cross-linking reaction between chemical groups or specific moieties between various polymeric networks is pivotal. The advancements in the synthesis of various polymeric NGs have unlimited biomedical applications, such as targeted drug delivery (TDD), gene delivery, diagnosis, imaging, cellular therapy, sensors, wound care, and tissue engineering, due to their different functionalization strategies [4]. Different biodegradable, biocompatible, and multi-stimuli-responsive biopolymers are used to impart fine-tunable functional characteristics to the NGs [5]. The evolution of macro-sized hydrogels to NGs has expansive potential applications, particularly in drug delivery, with the properties of sustained and/or controlled targeted drug delivery (TDD) to help to tackle the issues associated with adverse side effects of chemotherapeutic drug toxicity. In 1995, Doxil^®^ was the first FDA-approved antineoplastic nanodrug encapsulating doxorubicin (DOX) in PEGylated nanoliposomes for the passive targeting of tumors [6]. Three-dimensional (3D) printing has also been used to create 3D structural NGs. In 2015, using inkjet-based 3D printing technology, the first FDA-approved oral dosage form of the levetiracetam drug was produced, using poly(caprolactone) (PCL) and polyethylene oxide (PEO) biomaterials [7,8] (Table 1). In this review, we discuss the synthesis and functionalization strategies of NGs and the recent advances in the diverse biomedical applications of functionalized NGs.

## 2. Nanogels—Definition, Properties and Characterization

Generally, hydrogels are used as effective carriers in drug delivery systems (DDS) due to their physicochemical and biological characteristics for the delivery of drugs. With the advent of nanotechnology, the nano-sized/submicronic hydrogels (known as nanogels) with the range of 100–200 nm were developed for the optimum delivery of drugs at the target site due to their small size and swelling properties [9]. The term ‘nanogel’ (NG) or “nano-in-hydrogel” indicates colloidally stable nanosized hydrogel particles with large surface areas crosslinked, either physically or chemically, to incorporate cargo in the polymeric networks confined to a nanoscopic size. Vinogradov et al. [10] demonstrated a novel type of drug delivery system termed ‘NanoGel^TM^’ for hydrophilic particles, synthesized by cross-linking bifunctional networks of poly(ethyleneimine) (PEI) and carbonyldiimidazole-activated poly(ethylene glycol) (PEG) (PEG-cl-PEI) using an emulsification/solvent evaporation technique. The negatively charged antisense phosphorothioate oligonucleotides (SODN) specific to the human mdr1 gene were incorporated in NG particles for the effective inhibition of P-glycoprotein (P-gp) efflux pump expression in multidrug-resistant (MDR) human oral epidermoid carcinoma cells. NGs with specific functionality can change their stability in an environment-sensitive manner depending on the pH, temperature, and glutathione concentration [11].

NGs are three-dimensional (3D) hydrogels in the nanoscale regime, fabricated by crosslinking swellable polymers or polymeric nanocomposites, mostly in a spherical shape, to hold water without dissolution. Moreover, NGs have advantages over micron-sized hydrogels, with their large surface area-to-volume ratio, site specificity, tunable drug release, retention at the target site, drug loading, and release behavior, and gained increasing interest in diagnosis and therapeutic applications. Nevertheless, the removal of solvents and surfactants involved in the NGs synthesis, which is expensive, and the toxicity of the residual traces of polymeric monomers and surfactants, are disadvantageous. The unique properties of polymeric NGs are closely correlated to the fine-tuning of their size, porosity, amphiphilicity, stability, and charge from polymeric composition and the addition of functionalities by varying the chemical composition of the NGs [12]. Even though many carrier systems such as dendrimers, micelles, and drug conjugates are available, NGs are characterized by their improved colloidal stability due to lower rates of dissociation/degradation, higher retention of loaded cargo, and dispersion stability [13]. The engineering of NGs with the ability to encapsulate and carry cargo to specific sites and trigger selective responses with controlled drug release have made them an ideal material for TDD [14]. These engineered NGs become internalized by cells and deliver biotherapeutics in response to intracellular cues [15]. The incorporation of a plethora of components, such as inorganic nanoparticles (metal/metal oxide nanoparticles) and biomacromolecules (DNA, enzymes, proteins), brings versatility to NGs. The addition of inorganic nanoparticles with distinct properties such as magnetic, optical, and electrical conductivity to polymeric NGs produces a new class of agents known as “polymeric/inorganic nanohybrids”. Such nanohybrids have multi-functional activities, including diagnosis, imaging, and therapeutics [16,17].

The fabrication of NGs has developed various functional materials with the ideal size and multi-functionality for different fields of applications [18]. NGs have intriguing intrinsic characteristics such as high water content, biocompatibility, nanoscale dimension, and excellent water dispersibility, and the incorporation of different active bioactive ingredients, imaging dyes, and magnetic nanoparticles brings potential applications to NGs. However, for the in vivo applications of these NGs, the knowledge of the clearance during systemic delivery, the circulation half-time, and the factors affecting biodistribution and bioavailability is crucial. The interactions of the NGs with the biological components determine their biodistribution, retention time, and blood circulation times, which are influenced by the parameters such as size, shape, surface charge, hydrophilicity, surface modification (PEGylation), and functionalization with target ligands [19]. The surface modifications of NGs can be achieved with organic moieties to increase blood circulations, or for targeted/specific cellular uptake, or to avoid nonspecific uptake and delayed opsonization for various biomedical applications, ranging from drug delivery, imaging, and sensing to diagnosis and therapy. The polymeric NGs can undergo biological hydrolysis of esters and amides or reductive cleavage to yield side products, which need to be non-toxic. The U.S. FDA approved “Generally Recognized As Safe (GRAS)” molecules are generally used in polymeric NGs synthesis to avoid toxicity for biomedical applications. Furthermore, to minimize the toxicity and maximize the therapeutic effects, the payloads in the NGs need to be released at the target site without premature leaking while in circulation [20].

NG applications also depend on their particle size, swelling/de-swelling, surface charge, permeability, solubility, porosity, colloidal stability, drug loading/release capacity, response to stimuli, targeting, biocompatibility, biodegradability, and non-immunogenicity. A biodegradable and biocompatible NG with a size of 20–200 nm should avoid rapid renal clearance (elimination) and enter the reticuloendothelial system (RES). The interaction of functional groups with payloads determines the loading capacity of hydrophilic/hydrophobic payloads for NGs, and the rapid swelling/deswelling characteristics of NG without any immunological responses are pre-requisites for an ideal NG system [21]. Several techniques such as nuclear magnetic resonance (NMR) studies, Raman spectra, and Fourier transform infrared (FTIR) are used to characterize the crosslinking, molecular changes, and functionalities of polymeric NGs. The structural and morphological studies of NGs can be performed using microscopic techniques such as scanning electron microscopy (SEM), atomic force microscopy (AFM), and transmission electron microscopy (TEM), along with some scattering techniques, namely, dynamic light scattering (DLS), small-angle light scattering (SALS), small-angle X-ray scattering (SAXS), and small-angle neutron scattering (SANS) [22].

## 3. Stimulus-Responsive Nanogels

Materials that are able to trigger changes in its properties with external stimuli are called smart materials. Stimulus-responsive NGs are smart, intelligent, or environmentally sensitive nanostructures that exhibit sharp and evident changes, such as a changes in shape, volume, mechanical properties (by crosslinking/decrosslinking), and/or permeation rate in response to external or internal stimuli. These nanostructures can be used as smart systems for encapsulation, controlled drug delivery, gene delivery, imaging, immunotherapy, and theranostics. Smart stimulus-responsive systems provide a versatile platform for the integration and the controlled release of a variety of payloads, including drugs, proteins, and nucleic acids (DNA/RNA) materials in biological fluids. These NGs are mostly polymeric-based nanostructures that can be stimulus-responsive to environmental parameters, such as pH, ionic strength, mechanical stress, temperature, enzyme activity, redox potential, light, ultrasound, and magnetic/electric fields, for the subsequent structural and conformational changes in their polymeric network architecture, which result in the release of the active ingredients loaded in the NGs [23]. Stimulus-responsive NGs can be targeted to a specific cell type/tumor by coating nanostructures with ligands specific to the cell surface receptors or other specific interactions. These stimulus-responsive NGs are made to release the active cargo in the specific microenvironment of the tumor cells or intracellular spaces of cancer, or any cells, by responding to internal stimuli such as pH, temperature, redox potential, or other external stimuli [24,25]. These smart polymers can be made using natural/synthetic polymers or by incorporating a responsive functional group or compound along an existing polymer’s backbone. For instance, pH-sensitive bonds such as acetal, ortho ester, vinyl ester, and hydrazone encourage the controlled release of cargo under variations in pH values [26]. The anticancer drug DOX was conjugated to PEGylated nanoparticles via an acid-labile amide bond as a pH-triggered drug delivery system for lung cancer therapy [27].

## 4. Synthesis of Nanogels

NGs have superior swelling behavior along with important characteristics including high surface area, high colloidal stability, biocompatibility, high permeability, and biodegradability for enhancing high payload loading capacity with controlled/sustainable release capacity in targeted drug delivery systems. In addition, the features of NGs for administration (mucosal/parenteral), the release of hydrophilic and hydrophobic chemotherapeutic drugs, low immunogenicity, and ability to escape from the reticular-endothelial system (RES), such as macrophage cells, for longer circulation times can be tuned precisely through synthetic routes [28].

A variety of methodologies are used to synthesize NGs. In particular, microfluidics, inverse mini-emulsion, and inverse nanoprecipitation are important techniques used to synthesize NGs. Microfluidics have emerged as important, innovative approaches to manipulating small amounts of reagents with accurate control of mixing and physical processes at the microscale. In the microfluidic approach, glass capillary devices, or devices produced by the soft-lithography process, and polymers such as polydimethylsiloxane (PDMS) and poly(methyl methacrylate) (PMMA), are commonly used for microfluidic devices. By controlling the microfluidic conditions such as fluid rheology, flow rates, and chip design, it is possible to customize the size, dispersity, surface properties, payload delivery, and release profile [29]. Su et al. [30] synthesized doxorubicin-loaded manganese-alginate NGs (DOX@Mn-Alg) using a microfluidic chip for self-supplying hydrogen peroxide (H_2_O_2_) for synergistic chemodynamic therapy (CDT) and cancer immunotherapy. Alginate NGs synthesized with tunable pore size using a microfluidic approach were efficient in polypeptide/protein encapsulation and their sustainable release [31]. Similarly, lysozyme-loaded chitosan NGs with uniformity were continuously produced using a microfluidic coaxial flow reactor (CFR) [32]. Inverse miniemulsion is a water-in-oil (W/O) heterogenous polymerization technique that contains template nanodroplets formed by oil-soluble surfactants in a continuous organic medium. NG preparation by inverse nanoprecipitation involves the addition of an aqueous polymeric solution to a water-miscible non-solvent, resulting in the formation of nano-sized polymer clusters upon solvent and non-solvent mixing followed by crosslinking [33]. NGs are produced either with natural or synthetic polymers, or a combination thereof. However, NG formulation, composition and crosslinking can determine its applicability. NGs can encompass different inorganic components or moieties (ligands, antibodies, peptides) on the polymeric backbone for target imaging and therapeutic delivery through active/passive targeting to the desired site. For all strategies of NGs design, the parameters such as rheology, viscosity, and density need to be evaluated.

In addition to these classical methods, NGs can also be synthesized using recent innovative technologies such as PRINT (particle replication in non-wetting templates) and molecular imprinting. The lithographic technique is used in micro-/nanofabrication of precise and complicated 2D or 3D structures at extremely small scales [34]. Photolithographic techniques have been used to fabricate NGs by placing an ultraviolet (UV) cross-linkable polymer on a photoresist-coated wafer, pressed by a quartz template, and exposed to UV crosslinking with the removal of the quartz template. The fabricated NGs were further collected by the dissolution of the substrate in water. The PRINT technique helps to prepare monodisperse NGs, with control over composition, size, and shape [35]. DeSimone and coworkers fabricated monodispersed (200 nm) poly(ethylene glycol)-based (PEG) NGs with the PRINT methodology [36]. Using PRINT, Rolland et al. [12] fabricated monodisperse particles of poly(ethylene glycol diacrylate), triacrylate resin, poly(lactic acid), and poly(pyrrole), ranging from sub-200 nm to complex micron-scale objects, whereas molecularly imprinted polymeric (MIP) NGs are nano-dimensional and have imprinted molecular memory to address various biological issues. Cheubong et al. [37] prepared fluorescent serum porcine albumin-imprinted NGs (F-MIP-NGs), prepared via post-imprinting modification, for the potential application in the detection of pork contamination in halal meat extracts. Soluble MIP-NGs (17 nm, 97 kDa) of synthesized synthetic antibodies with dimensions and molecular weight mimicking those of real biological antibodies showed good affinity and selectivity for the target [38]. Very recently, MIPs of synthetic antibodies for protein recognition have been synthesized by the epitope approach [39].

## 5. Crosslinking of Nanogels

The synthesized/fabricated NGs are further crosslinked based on physical (non-covalent) or chemical (covalent) crosslinking to provide stability to nanostructures. Physical crosslinking is a weak interaction (often reversible) between polymeric chains formed under mild conditions. It is stabilized by relatively weak interactions, such as electrostatic interactions, Van der Waals forces, hydrogen bonds, and hydrophilic/hydrophobic or host-guest interactions between polymeric networks [33]. These kinds of reversible non-covalent crosslinks are formed with the conjugation of amphiphilic blocks, self-assembly, agglomeration of polymeric networks, and in the incorporation of oppositely charged polymeric networks. Self-assembled micellar NGs produced with linked copolymers in an aqueous solution, or assembly of amphiphilic blocks with hydrophilic shell and hydrophobic core, are used to encapsulate payloads for drug delivery and other applications [40].

On the other hand, chemical crosslinking (often irreversible) is stronger and more stable compared with physical crosslinking. The commonly employed method for chemical crosslinking is the formation of a covalent bond between the polymeric chains by heterogeneous polymerization reactions in the presence of bifunctional or multi-functional crosslinkers [41,42]. The use of properly selected cross-linking agents and the appropriate use of their quantities allows for tuning the mesh size, the degree of crosslinking, morphology, and the dimension of NGs, which are crucial parameters for use in desired applications. These characteristics improve the colloidal stability and limit the leakage of the payload by unwanted dissociation of the polymeric network under in vitro and in vivo conditions. Many cleavable linkers are covalently bonded and responsive to a wide range of stimuli, such as pH, light, temperature, enzyme, redox, hypoxia, and electric and magnetic fields. Liposome-coated NGs with combined features of both liposomes and core covalently crosslinked NGs are considered an important drug delivery platform for polymer hydrogel–lipid hybrid nanocarriers, which can be able to balance extracellular stability and intracellular drug release to achieve high therapeutic efficacy and low side effects [43,44]. Covalent crosslinking can also be performed in the polymerization of low molecular weight monomers, as well as covalent coupling of the reactive functional groups of macromolecular precursors to form NG networks, which allows tuning the properties and structure of the gel particles [45].

Several crosslinking techniques, such as radical polymerizations, click chemistry approaches, Schiff-base reactions, thiol–disulfide exchange, amide bond formation, photo/thermally-induced crosslinking, and enzyme-mediated crosslinking, have been devised for the synthesis of stable NGs from polymeric precursors [13]. The factors influencing the particle size in the synthesis of NGs are the polymer concentration and environmental parameters, such as pH, temperature, and ionic strength. Irradiation-induced synthesis of protein NGs via gamma irradiation of bovine serum albumin (BSA) was also demonstrated [46,47,48]. Even controlled radical polymerization (CRP) techniques such as atom transfer radical polymerization (ATRP), reversible addition/fragmentation chain transfer polymerization (RAFT), and nitroxide-mediated polymerization (NMP) were used to prepare NGs with different 3D composition and architectures (core-shell/hollow) [48,49]. The combination of radiation-induced synthesis with RAFT achieved the synthesis of NGs with narrow molecular weight distributions, controlled molecular weights, and complex architectures. Kirac and Guven [50] synthesized poly(N-isopropylacrylamide) (PNiPAAm) by RAFT-mediated gamma-radiation-induced polymerization with narrow dispersity. Overall, developments in polymer chemistry, crosslinking, and manufacturing techniques have resulted in extraordinary diversity and control over the composition, architecture, and functionalization of NGs, which in turn gives more possibilities in the synthesis of desired NGs with novel characteristics required for specific biomedical applications.

## 6. Nanogel Functionalization Strategies

**Figure 1:**
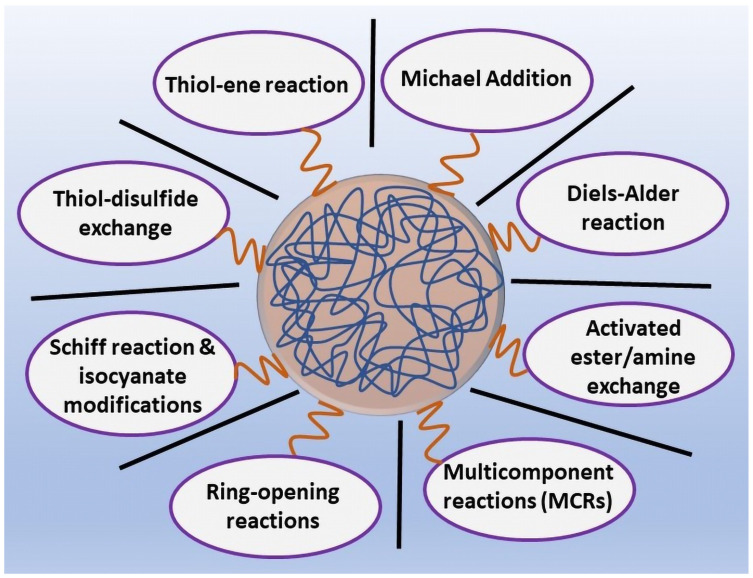
Various functionalization strategies involved in the synthesis of functionalized NGs.

One of the most significant characteristics of NGs is the ability to functionalize their structure with various polymers, molecules, or targeting ligands/moieties for their specific applications. Notably, surface functionalization and surface properties are important in influencing the final characteristics of the NGs. Crosslinked NGs with a high-surface area and good structural stability are important parameters for functionalization [51]. The introduction of chemical moieties to NGs can be achieved though chemical or physical linkages. Their functionalization can be performed either by synthesizing NGs using polymerization of monomers with functional moieties that remain as reactive groups for the selective transformation into other functional groups, or by post-polymerization modifications (PPMs), where the chemical modification of NGs is performed after the polymerization under very mild conditions. The various chemoselective coupling strategies used to synthesize functional polymers by PPM are mentioned below [52] (Figure 1).

### 6.1. Activated Esters/Amine Chemistry

Activated ester–amine chemistry is a classic example of a PPM chemical reaction. Under mild reaction conditions (catalyst-free, room temperature), the activation of esters to form amide bonds represents one of the most interesting strategies for polymeric NGs functionalization. It is advantageous, owning to the abundant availability and accessibility of amines from natural/synthetic sources [53]. Esters have emerged as some of the most flexible moieties for polymer functionalization, and the amides are very versatile linkages in organic chemistry, exhibiting unique stability towards extreme chemical environments [54].

### 6.2. Click Chemistry

Click reaction is based on utilizing rapid reactions with the potentiality to greatly facilitate the development of covalently functionalized nanostructured biomaterials for biomedical applications [55]. The Diels–Alder (DA) [4 + 2] cycloaddition is one of the click reactions that do not require any metal catalysts, and occurs under mild conditions. It is stereoselective, highly efficient, and gives high yields, and the reaction occurs between a conjugated diene and a substituted alkene (dienophile), during which six π-electrons rearrange to form a cyclic, six-membered product [56]. Copper(I)-catalyzed (CuAAC) or copper-free strain-promoted azide–alkyne cycloadditions are also performed. Functionalization with azide and alkyne groups offers opportunities to conjugate different biomolecules to polymeric surfaces in the nanosystem. In addition, Michael additions, thiol–ene reactions, and oxime reactions also follow click chemistry, ensuring high yields, regiospecificity, and fast kinetics, to generate nanomaterials with diversity and improved properties [57].

### 6.3. Thiol Chemistry

Thiol chemistry occurs in functional thiols and participates in bioconjugation techniques. It includes free radical thiol–alkene/alkyne reactions, thiol–disulfide exchange, thiol–Michael addition, and thiol–actone double modification [13]. In radical- or light-mediated thiol–ene/thiol–yne coupling reactions, the thiol reacts with a wide variety of unsaturated functional groups (maleimides, acrylates, and norbornenes) in addition to unactivated carbon–carbon double/triple bonds, satisfying all the criteria for being a click reaction, to be combined with those of the photoinitiated process, which can be activated at specific times and locations for the synthesis of tailorable nanomaterials [58]. The advantages of this reaction are its simplicity, high yields and conversion, high reaction rates, and the possibility of photoinitiation [59]. Generally, the thiol–disulfide exchange reaction is an S_N_2-type nucleophilic substitution of a thiol in disulfides with another thiol. Even oxidation of free thiol groups can form disulfide bond formation [60].

### 6.4. Isocyanate Modifications

Isocyanates are well-known to react efficiently with thiols, amines, and alcohols under mild conditions without any side products for polymer nanomaterials [61]. Post-polymerization functionalization of hydroxyl-group terminated polymers, such as poly(ethylene glycol), poly(N-isopropylacrylamide), poly(N.N-dimethylacrylamide), and poly(tert-butyl acrylate), with a wide variety of functional aromatic and aliphatic isocyanates, can react with primary amines, thiols, or alcohol nucleophiles as side chains of functionalized polymers [62]. However, these applications are restricted by the toxicity and instability or sensitivity of isocyanate-containing polymers with potential decomposition in moisture [63].

### 6.5. Schiff Reaction

Schiff base linkages are one of the highly efficient post-polymerization functionalizations of polymers and biological macromolecules. It includes imines, hydrazones, and oximes, which are the products of condensation reactions between aldehydes/ketones and primary amines, hydrazides, and aminooxy functional groups, respectively [64,65]. Compared to imines, oximes and hydrazones are chemically stable with pH values. These linkages are excellent due to their pH-sensitivity, reversibility, and biocompatibility, which offers an excellent nanomaterial response over biologically relevant stimuli [66].

### 6.6. Michael Addition Reaction

Michael addition is a special type of 1,4-addition of carbon–carbon (C–C) bond formation reaction, in which the nucleophilic addition of a carbanion enolate (Michael donor) adds to an α,β-unsaturated carbonyl compound (Michael acceptor) containing an electron-withdrawing group. The thiol–Michael addition reaction is the intersection of the thiol–click reaction and Michael addition reactions [67]. The thiol–Michael addition click reaction produces highly stereospecific and regiospecific products. In the thiol–Michael addition reaction, the “ene” is electron-deficient (maleimides, α,β-unsaturated ketones, fumarate esters, acrylonitrile, cinnamates, and crotonates) and the reaction is initiated using catalysts, including strong bases, metals, organometallics, and Lewis acids [68]. In the thiol–maleimide click reaction, triethylamine was used as a basic catalyst to functionalize degradable polyesters [69,70]. The thiol–Michael addition reaction offers an enhanced level of control over reaction parameters to ensure both spatial and temporal modification to nanomaterials. Both the nucleophile- and base-catalyzed thiol–Michael addition mechanisms do not lead to the formation of significant side products, such as the radical–radical termination products formed in radical-mediated thiol–ene reactions [59,67,71].

### 6.7. Ring-Opening Reactions

Ring-opening reactions are extremely versatile processes in polymer science. The strained heterocycles (epoxides, aziridines, and azlactones) undergo ring-opening reactions with strong or weak nucleophiles (amines, azides, alcohols hydroxides, thiols, cyanides, Grignard reagent, and lithium aluminum hydride (LiAlH_4_)) enabling the introduction of desired heteroatoms on the polymer backbone [72].

### 6.8. Multi-Component Reactions

Multi-component reactions (MCRs) are an important repertoire of methodological strategies for synthetic and polymer chemistry [73]. It is a one-pot synthetic process that produces a single product from three or more reactants through a cascade of elementary reactions, in a highly regio- and stereoselective manner, to form complex organic molecules [74]. These kinds of reactions include isocyanide-based MCRs, free-radical mediated MCRs, and MCRs based on organometallic compounds, as well as metal-catalyzed MCRs. The unique feature of MCR includes the synthesis of highly functionalized molecules for biological interests [75].

For biomedical applications, these biorthogonal strategies help to make the covalent bond between various biological moieties and the natural/synthetic polymeric network, linking targeting ligands, chemotherapeutic moieties, imaging agents, and diagnostic sensors. Physical modifications of NGs can be achieved by non-covalent interactions, including hydrogen bonding, charge transfer interactions, van der Waals, and polyelectrolyte complexation. In the case of physically crosslinked NGs, such as the self-assembly of amphiphilic polymers along with encapsulating agents, the conjugation of a polymeric network with desired functionality needs an adaptable nanocarrier. Akiyoshi described the first physically cross-linked NGs using the self-assembly of cholesterol-bearing polysaccharides in an aqueous solution, and used as nanocarriers for drug delivery [76]. Self-assembly of maleic anhydride functionalized chitosan and bovine serum albumin (BSA) proteins yielded NGs through the interaction of macromolecular compounds to form polyelectrolyte complexes. These self-assembled NGs provide pH- and temperature-sensitivity for DDS [77].

## 7. Biomedical Applications of Functionalized Nanogels

**Figure 2:**
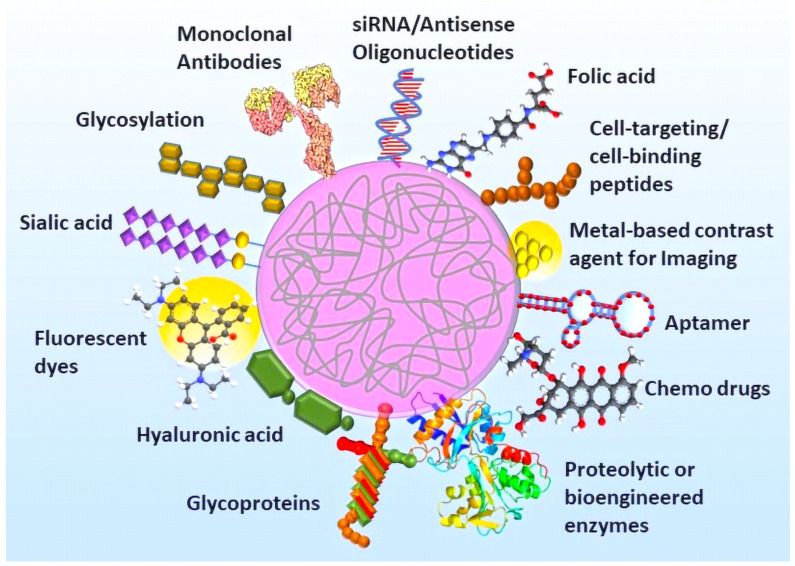
Surface modifications of NGs with various chemical/biological moieties for biomedical applications.

NGs are promising nanosized hydrogel systems with the properties of hydrogels and nanoparticles. They are tunable in size, surface modifications, and/or chemical functionalization, and stimuli-responsiveness to various factors depending on the desired applications. For biomedical applications, most of these nanostructures lack cellular specificity, and indiscriminately circulate in the blood in large amounts for their activity, causing adverse side effects. Therefore, surface functionalization of NGs is greatly important to enhance cell targetability, avoid macrophage uptake, reduce systemic toxicity of chemotherapeutic drugs on normal cells/tissues, and reducing unwanted interactions with biological barriers. A myriad of moieties targeting cell receptors, antigens, biomolecules, nucleic acids, and lectins are generally surface-functionalized for specific cell targeting. In amphiphilic self-assembled polymeric NGs, hydrophobic core functionalization shows advantages in the incorporation of hydrophobic payloads for the stimuli-responsive controlled drug/biomolecule release (Figure 2).

### 7.1. Functionalized NGs in Cancer Theranostics

Cancer theranostics combine both cancer therapy and diagnosis for concomitant early diagnosis with molecular imaging for spatiotemporal tumor treatment (Table 2). The use of protein-based NGs has many advantages, such as non-antigenicity, biodegradability, and biocompatibility, with great options for surface modifications and chemical conjugations. Protein NGs from bovine serum albumin (BSA), which is a natural transporter of hydrophobic molecules such as vitamins, hormones, and many plasma components [78], have been greatly used in the delivery of therapeutic molecules. However, protein NGs are highly susceptible to in vivo proteolytic degradation by enzymes at physiological pH conditions, which results in the premature release of therapeutic molecules. To address this challenge, protein–polysaccharide conjugates are formed by the covalent conjugation of polysaccharides to the protein NGs, via the Maillard dry-heat reaction, through the linkage between the reducing carbonyl group of polysaccharides and the ε-amino groups in the protein [79], followed by the thermal-gelation processes to form a core-shell structure. To target these protein-carbohydrate NGs (core/shell) to a particular cell-type, cell-specific targeting moieties such as folic acid (FA), which is overexpressed in a large number on tumors, can be spatially positioned on the polysaccharide shell. Molecular modeling studies with other experimental approaches showed that FA affects the enzymatic activity and enzyme conformational structures of proteolytic digestive enzymes, such as α-amylase, pepsin, and trypsin, thereby protecting the FA-functionalized protein–polysaccharide NGs from proteolytic degradation [80]. Borah et al. [81] reported the design of novel BSA-FA functionalized amylopectin (BSM) NGs with core-shell conformation with encapsulated curcumin. These NGs were resistant to in vitro oral-gastrointestinal digestion and cell-specific internalization in the human HT29 colon cancer cells via folate-receptors (FR). Photothermal therapy (PTT) is one of the important noninvasive, high-accuracy cancer treatment methods to efficiently inhibit the development of tumors. PTT employs the heat energy generated from the NIR radiation to destroy tumor cells. NIR regions are comprised of NIR-I (700–950 nm) and NIR-II (1000–1700 nm) regions. In comparison to NIR-I, NIR-II is efficient in PTT due to its relatively high maximum permissible exposure (MPE), deep penetration into tissues, and less photon scattering and tissue interaction [82], whereas magnetic resonance (MR) imaging uses a strong magnetic field and radio waves to make 3D images of cross-sections of tissue structures without radiation. For efficient monitoring and therapeutic outcomes, it is necessary to incorporate imaging agents with photothermal agents. The incorporation of both photothermal and MR contrast agents in a single nanoplatform can provide imaging-guided tumor PTT. Zhang et al. [83] prepared Gd/Cus@PEI-FA-PS NGs containing polyethylenimine (PEI) NGs with surface functionalized Gd(III) chelates and FA targeting ligands, along with in situ formations of CuS NPs as nanoprobes, for specific MR and photoacoustic (PA) dual mode imaging, along with high photothermal conversion efficiency (PCE) for PTT on a xenografted tumor model. In photodynamic therapy (PDT) of anticancer treatment, the therapeutic effect of PDT diminished in the hypoxic environment of solid tumors. Thus, PDT uses a specific wavelength of light to activate a photosensitizer to produce reactive oxygen species (ROS) during oxygen reduction [84]. The development of a self-fluorescent hyaluronan–fullerene (C_60_)/TPENH_2_ NG loaded with tirapazamine (TPZ) with AIE characteristics produced ROS under laser irradiation for anticancer activity through synergistic PDT and hypoxic-activated bioreduction therapy (BRT) [85]. The oxygen consumed in the PDT process provides an anoxic internal environment for the production of cytotoxins by the activated TPZ drug for the synergistic anticancer activity in the CD44-targeted delivery. These NGs with TPENH_2_ provide AIEgen characteristics that were self-fluorescent for cell imaging applications.

The nanosystem needs to discern between the healthy and cancer cells for therapeutic efficacy without side-effects to release chemotherapeutic agents in a controlled way. A biocompatible, carboxyl-functionalized polyvinylpyrrolidone (PVP-co-acrylic acid) NG was synthesized by e-beam pulsed irradiation and conjugated with tumor cells targeting ligand FA to recognize FR. These NGs were also conjugated with anticancer DOX or to a pro-apoptotic Bcl-2 siRNA by GSH-responsive redox-sensitive spacer, 3-(2-aminoethyl) dithiopropionic acid (AEDP), to obtain a controlled release mechanism specific for cancer cells through FR targeting in a co-culture system of HeLa (a positive FR, cancer cell line) and NIH3T3 cells (a negative FR, fibroblast cell line) [86]. Similarly, a glycol chitosan NG decorated with folate to target FR was internalized through flotillin-1 and Cdc42-dependent endocytosis, and the uptake of the NG requires the involvement of the actin cytoskeleton via macropinocytosis. These NGs have great potential as DDS for targeting different intracellular compartments [87].

Many cancer therapy drugs, including doxorubicin, cisplatin, and 5-fluorouracil, are unable to overcome multidrug resistance (MDR) in solid tumors in clinical treatment. However, the effective accumulation of therapeutic drugs and an increase in the efficacy of anticancer agents can bring the solution. Moreover, the drug carriers also need to be designed to overcome the drug efflux and enhance the inhibitory activity on MDR solid tumors. For cancer therapy, cisplatin, a metal platinum(II) (Pt(II)) drug, has been widely used for several decades, which inhibits DNA synthesis and induces free radical-induced cell damage or apoptosis [115]. However, the therapeutic efficiency of Pt(II) is limited due to limited accumulation at the tumor region and inefficient internalization by tumor cells. Moreover, the in vivo non-specific uptake of platinum drugs causes severe systemic toxicities, including ototoxicity, nephrotoxicity, myelosuppression, and allergic reaction [114,116]. Compared to other Pt(II)-based chemotherapy, oxaliplatin (OXA) has higher efficacy and lower toxicity [117]. Very recently, FA-conjugated HA-coated alginate (AL) NGs with loaded OXA (F/HA/AL/OXA) were prepared by the crosslinking process for the treatment of colorectal cancer. These NGs showed enhanced antitumor activity on the HT29 cell line via CD44 receptor-mediated endocytosis with the upregulated expression of the apoptotic gene *Bax*, along with the down-regulated expression of anti-apoptotic gene *Bcl-2* [88].

Despite accumulating Pt(II)-based nanocarriers at the tumor site, the intrinsic and acquired MDR of tumors causes the efflux of Pt(II)-based drugs, and intracellular glutathione (GSH) easily chelates Pt(II) to form water-soluble Pt-GSH, which is excreted from tumor cells, thereby inhibiting the formation of adducts/crosslinks with DNA purine bases, with a preference for guanine, and not impeding genome replication, transcription, and triggering cell apoptosis [118]. To tackle these problems, recently, it was found that phenylboronic acid (PBA) and its derivatives, which have higher selectivity in recognition and binding to sialic acid (SA) residues exposed on the surface of cancer cell membranes, can be targeted by nanocarriers [119]. Xu et al. [114] developed a smart, sequentially responsive NG (~160 nm), mPOE–PBA–PAPE/Pt, by co-polymerization of methacrylate phenylboronic acid (APBA) and its ester derivative (MAPAPE), Pt(IV) crosslinker, and slightly acid-sensitive polyethylene glycol derivative (mPOE), to improve in vivo selective drug delivery and GSH-mediated platinum resistance. Previously, α-tocopherol polyethylene glycol 1000 succinate (TPGS)-based drug nanocarrier, which effectively interferes with the fluidity of cell membranes and the function of mitochondria, was used to reverse drug efflux in MDR cancer therapy. Bao et al. [99] developed a pH-sensitive prodrug, TPGS-CH=N-Dox, by conjugating DOX onto TPGS via a cleavable Schiff base linkage. This prodrug was mixed with PEGylated lipid to form a multi-functional hybrid micelle system for high drug loading capability and extended circulation time related to the inhibition of drug resistance in MDR cancer cells, and improved therapeutic response in the MCF-7/ADR cell and mouse liver cancer model. However, the Schiff base linkage failed to cleave rapidly under the mildly acidic conditions of endo/lysosomes in cancer cells. Therefore, the combination of a novel acid-labile TPGS and an *ortho* ester linkage in the nanoplatform can increase drug retention in MDR cancer cells. An ortho ester-conjugated TPGS (T-OE) was grafted onto diallyl disulfide (DADS)-crosslinked NGs (TNG) to fabricate dual-functionalized NGs with DOX loading to enhance the inhibitory effect on (MCF-7/ADR) MDR cancer cells via the combined activities of anticancer drug activity, increased ROS concentration, and the ability to overcome cell reflux effectively by inducing mitochondrial depolarization and interfering with the ATP metabolism [120].

The multiple response hybrid NGs can be manipulated in tandem with exogenous and endogenous activation to address the complexity of biological systems. Remote optical sensing and drug delivery using an environmentally guided, magnetically driven hybrid NG particle could allow for cancer diagnostics and treatment. Wu et al. [121] designed a multi-functional core-shell nanostructured hybrid NG (<200 nm) by synthesizing magnetic nickel nanoparticles (Ni NPs), followed by the growth of fluorescent silver nanoparticles (Ag NPs) on the surface of Ni NPs to form Ni–Ag bimetallic core-shell structures (18 ± 5 nm) with immobilization of pH-responsive p(EG-MAA) gel shell on the Ni-Ag NPs. This class of hybrid NGs can enter cells intracellularly, overcoming the cellular barrier, and fluoresce in mouse melanoma B16F10 cells. Further, the remote magnetic guidance with biochemical sensing can deliver the anticancer drug 5-fluorouracil by a pH-regulated delivery system.

Reductive tumor microenvironments trigger responsive nanosystems for controlled drug delivery. GSH is 10-fold higher in cancer lesions than in normal cells [94]. HA is a major constituent of the extracellular matrix (ECM) and synovial fluid, and also has several advantages, such as biocompatibility, anti-adhesivity, biodegradability, and non-immunogenicity for biomedical applications [122]. Thus, HA can be used as a targeting ligand for the delivery of various molecules. Tocopherol succinate (TOS) grafted HA, via disulfide bonds to obtain HA-ss-TOS conjugate, was assembled into nano-micelle. Reductive-responsive paclitaxel (PTX)-loaded HA-ss-TOS-PTX was efficiently taken up by CD44-induced endocytosis on CD44 overexpressing B16F10 melanoma cells, and exhibited enhanced cytotoxicity [94]. Self-monitoring multimodal synergistic theranostic strategies in multi-responsive tumor microenvironments significantly improve therapeutic efficacy. Designing small fluorescent HA NGs, which can respond to reducing the tumor microenvironment and the controlled release of therapeutics through active targeting with light-traceable monitoring, is a promising theranostic nanoplatform. Therefore, a novel multi-functional HA NG by encapsulating gold clusters and DOX for combined diagnosis and therapy was developed. These mHA-gold cluster (mHA-GC) NGs were conjugated with disulfide linkage, which can readily disassemble in response to the reductive environment with the release of entrapped DOX. More importantly, mHA-GC NGs can emit NIR fluorescence signals to detect tumor areas and monitor the DOX drug delivery by in vivo imaging in the reductive tumor microenvironment [91]. Ma et al. [95] prepared multi-functional self-fluorescent HA-based NGs (HNPs) (HA-ss-ATRA/TPENH_2_) using all-trans retinoid acid (ATRA)/aggregation-induced emission luminogen (AIEgen) fluorophores (TPENH_2_)-grafted HA with disulfide bonds as redox-responsive linkers for DOX delivery. As expected, DOX-loaded HNPs rapidly disintegrated in the tumor microenvironment (20 mM GSH), and the self-fluorescent DOX-loaded HNPs with unique AIEgen characteristics become internalized into the cytoplasm through passive and active targeting via CD44 or LYCE-1 receptors. This novel cancer-targeted delivery system for the controlled release of drugs for enhanced antitumor efficiency also provides real-time intracellular imaging. The use of nanotechnology in drug delivery is a promising approach for improving the therapeutic index and dose-limiting side effects associated with payloads. Among several Food and Drug Administration (FDA) approved DDS, Doxil (liposomal doxorubicin), SMANCS (polymeric conjugate of neocarzinostatin), and Abraxane (albumin-bound paclitaxel nanoparticles) are the most prominent [123,124,125]. All the nanoparticle formulations rely on the EPR mechanism, which is a passive targeting process, thus incorporating targeting ligands on the surface of nanoparticles enables active targeting of the antineoplastic tissues, improving the therapeutic index by reducing toxicity. The combination of liposome and NG offers great advantages for surface functionality on the shell, and stable drug loading in the core. The lipid-coated NGs use a lipid bilayer as a template to control the formation of a cross-linked polymeric core. The targeting ligands or other functionalization moieties are incorporated into the lipid bilayer, and the cross-linked polymeric core acts as a carrier of drug cargo. Murphy et al. [126] described lipid-coated NGs with the targeting ligands for the encapsulation of a wide array of monomers, with photo-crosslinking for the delivery of drug chemotypes, and imaging agents for theranostic applications.

**Figure 3:**
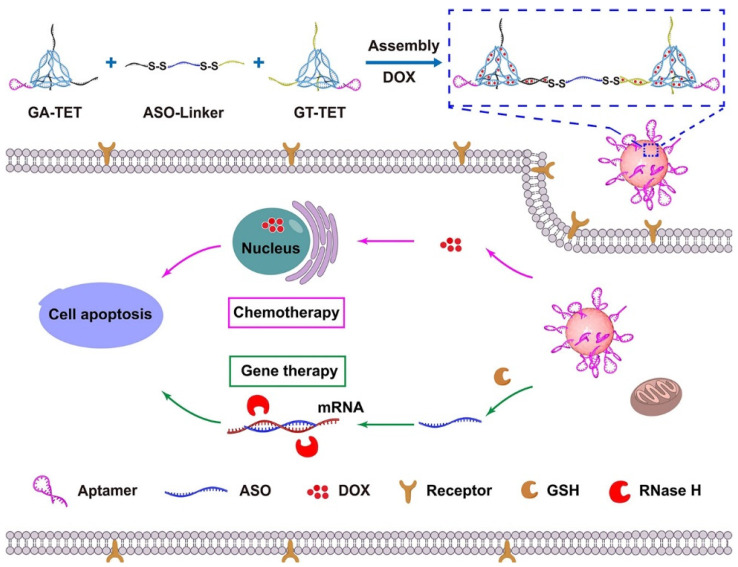
An aptamer-modified DNA tetrahedron-based NG for combined chemo/gene therapy of MDR tumors. Reproduced from Tang et al. [111] with permission from American Chemical Society, USA.

Cancer chemotherapy in MDR cells is a big challenge, and the knockdown of MDR-associated genes by gene silencing is a promising solution to improve the chemotherapeutic effects of anticancer drugs in MDR tumors. Various delivery systems such as liposomes, polymers, hydrogels, dendrimers, or inorganic host systems have been used to co-deliver antisense oligonucleotides, small interfering RNA (siRNA), or short hairpin RNA (shRNA) for gene silencing, and chemodrugs for combined tumor therapy [127]. DNA nanotechnology provides various 2D and 3D structures in different sizes and shapes, such as tetrahedron, triangular prism, icosahedron, and DNA origami structures in bottom-up fabrication strategy, and aptamers are artificial short oligonucleotide DNA or RNA sequences (25–80 bases) that bind a specific target molecule. The fabrication of an aptamer-modified DNA tetrahedron (TET)-based NGs was used for the combined chemo/gene therapy of MDR tumors. These multi-functional DNA-based NGs were site-specifically functionalized with targeting ligands (anti-MUC1 aptamer) to target O-glycosylated protein Mucin 1 (MUC1) overexpressing human MCF-7 breast tumor cells with DOX resistance (MCF-7R), and provides controlled-release elements (GSH-responsive disulfide bridges) for the co-delivery of DOX chemodrugs and gene therapy drugs [111] (Figure 3). Recently, poly(lactic acid)-PEG-Apt/Dox NPs have also developed as a tumor-targeting delivery system based on MUC1 aptamer-targeted nanoparticles for anticancer activity on the MUC1-overexpressing A-549 cell line (human alveolar basal epithelial cells) [112]. In another instance, nanomedicine in treating high-grade gliomas crossing the blood-brain barrier (BBB) by passive targeting through the EPR effect has limited drug therapeutic efficacy. The high-grade gliomas can easily infiltrate the surrounding brain tissue, and thus tumor-specific drug delivery across the intact BBB is required. Therefore, the DDS of therapeutic substances should have the ability for an efficient and selective crossing of the BBB, evading the efflux pump, and should have good stability from degradation. All these characteristics of the DDS are solely dependent on their physicochemical and biomimetic features, irrespective of their cargo structure. Glioblastoma, a fatal form of brain tumor, is the most aggressive tumor and rarely metastasizes. A stimuli-responsive NG crosslinked via a matrix metalloproteinase (MMP-2/9) substrate and armed with 5-[^125^I] iodo-4′-thio-2″-deoxyuridine ([^125^I]ITdU) (Auger electron-emitting drug), and post modified by the surface functionalization with diphtheria toxin receptor (DTR) ligand cross-reactive material 197 (CRM-197), was fabricated for allowing the transcytosis across the BBB at the tumor site. At the tumor site, the upregulated expression of MMP2/9 degrades these NGs and releases [^125^I]ITdU, which gets incorporated into the DNA of glioblastoma cells. This strategy efficiently delivered radiopharmaceuticals intracellularly across the BBB for glioblastoma therapy [103]. Despite total resection, glioblastoma invariably recurs within 2 cm of the resection cavity among patients [128]. Immunotherapy is an effective alternative treatment strategy to gene and chemotherapies, where tumor cells residing beyond the resection cavity can be targeted without damaging the healthy normal brain tissue, by transferring anti-glioblastoma adoptive T lymphocytes toward a selective tumor cell-surface antigen [129]. Chitosan shares structural similarities with glycosaminoglycans (GAGs) of native ECM, and the development of biodegradable, thermoreversible poly(ethylene glycol)-*g*-chitosan (PCgel) hydrogel acts as a depot for locally sustained delivery of therapeutic cytotoxic T lymphocytes (CTLs) to glioblastoma cells for brain tumor immunotherapy [92].

Many MDR tumors and cancer stem cells (CSCs) overexpress elevated levels of the CD44 receptor, which binds to hyaluronic acid (HA). The synthesis of an NG–drug conjugate that exerts CD44 receptor-targeted activity suppresses MDR tumors/CSCs based on membranotropic activity. The cholesteryl-HA (CHA) with a hydrophobic core and high drug loads (up to 20%) (CHA-drug NG) demonstrated a sustained drug release by the hydrolysis of the biodegradable ester linkage. These NGs demonstrated higher cytotoxicity in CD44-expressed MDR human breast and pancreatic adenocarcinoma cells by internalization of NGs via CD44 receptor-mediated endocytosis, and by the interaction with the cancer cell membrane. These CD44 targeting strategies using HA-based therapeutic NGs can significantly enhance drug (etoposide, salinomycin, curcumin) bioavailability, and treat MDR cancer cells and multicellular spheroids [89]. Similarly, using curcumin (CUR) as a natural anticancer and anti-inflammatory compound, NGs were synthesized. CUR was conjugated as an ester to CHA (CHA-CUR) to prepare the NGs for the targeted delivery of Cur to CD44-expressing MDR cancer cells. CHA-CUR NGs showed targeted accumulation and effective tumor growth inhibition in human pancreatic adenocarcinoma MIA PaCa-2 cell line in vitro, and aggressive orthotropic murine mammary carcinoma 4T1 in an animal model. It induced apoptosis in cancer cells, suppressing the expression of nuclear factor-κB (NF-kB), tumor necrosis factor-alfa (TNF-α), and cyclooxygenase 3 (COX-3), of molecular targets similar to free CUR [90].

Nucleic acid-based gene therapy offers promise for many genetic diseases. For nucleic acid delivery, non-viral gene carriers such as polymeric systems are generally used as promising gene vectors, but they demonstrate poor transfection performance even though they are finally removed from the body circulation. Generally, high molecular weight polycationic polymeric vectors have better transfection performances, however, linear poly(glycidyl methacrylate) PGMA-based vectors were non-degradable and had relatively higher toxicity. Low-molecular-weight polyethylenimine (PEI)-based NGs with biscarbamate crosslinking demonstrated enhanced transfection activities with no cytotoxicity [130]. NGs are specifically targeted by the conjugation of biomolecules guiding to a specific cell/tissue type. Recently, low-molecular-weight PGMA was used as a potential nucleic acid vector. An ethylenediamine (ED)-functionalized low-molecular weight PGMA (PGED) with reducible (disulfide linkages), cationic NGs were prepared by crosslinking of PGED with α-lipoic acid (LA), which was performed by simple reduction and oxidation of lipoyl groups for highly efficient delivery of plasmid DNA (pDNA) and siRNA to hepatoma cells, suppressing cancerous cell proliferation and migration [110]. Similarly, carboxyl functional NGs were prepared by the pulsed electron-beam irradiation (40 kGy)-induced crosslinking of semi-dilute poly(N-vinyl pyrrolidone) (PVP) aqueous solution in the presence of acrylic acid. The pendant carboxyl groups of the PVP NGs were conjugated with the terminal amino group of the antisense oligonucleotides (ODN), forming amide bonds, and this system was used as a nanocarrier for intracellular delivery of genetic materials for therapeutic applications [109].

For efficient anticancer activity, the nanomedicine should penetrate the solid tumor tissue sufficiently. A novel biodegradable cancer therapeutic nanoplatform was developed with improved tumor permeability for the selective and controlled delivery of anticancer agents, with the ability to release ultrasmall nanoblocks in the tumor microenvironment. Zhou et al. [98] prepared positively charged NGs with double-crosslinking of chitosan with an ionic physical gelator (sodium tripolyphosphate) and a disulfide-containing chemical crosslinker. After assembly with an anionic oligomer of poly(acrylic acid), the cationic NGs were transformed into negatively charged nanocarriers (CTCP) for the effective encapsulation of cationic anticancer agent DOX to generate DOX@CTCP, for encapsulating pDNA through electrostatic interactions. DOX-loaded CTCP is cleaved under a reductive tumor microenvironment of elevated GSH and lysozyme to release ultrasmall nanovesicles. Along with the use of galactose as a functionalized targeting ligand to the hepatocellular carcinoma (HCC) site, oridonin (ORI)-loaded self-assembled galactosylated chitosan-*graft*-poly(N-isopropylacrylamide) (Gal-CS-*g*-PNIPAm) pH-sensitive NG was used as a targeted nanocarrier for the HCC. The release behavior of ORI from NGs was pH-dependent, and the anticancer activity increased with an increase in the number of galactose moieties on the NGs in HepG2 cells (liver cancer cells). This demonstrated that these NGs enhanced the active uptake of ORI into liver cancer cells via asialoglycoprotein receptor-mediated endocytosis through active-targeted drug delivery [96]. Similarly, the glucose and maltose-bearing thermosensitive poly(N-vinylcaprolactam) (PNVCL) soft NGs in a semi-batch precipitation polymerization were prepared. Targeting these NGs to C-type lectin (CTL) receptors causes preferential uptake by the macrophages/dendritic cells and improves the cross-presentation of antigens for tumor immunotherapy. Initially, propargyl acrylate (PA), a comonomer, was used to introduce the terminal alkyne functional group on the PNVCL NG surfaces (PNVCL–PA). The alkyne group was further modified with different azido-glucosides and -maltosies via the CuAAC “click” reaction. Carbohydrate-functionalized PNVCL NGs are more swollen and hydrophilic, having the potential as DDS, and maltose derivatives exhibited stronger binding to model lectin concanavalin A (Con A) [97].

Multivalency is an important property of polymeric nanocarriers for efficient biomedical functioning. Targeted ligands including antibodies, aptamers, or peptides have been used to decorate nanocarriers to achieve enhanced tumor accumulation. T-cell activation is only possible with the simultaneous ligand binding to the co-stimulatory molecules CD3 and CD28 via monoclonal antibodies (mAbs). However, these mAbs in the solution failed to trigger T-cell activation. The dendritic polyglycerol (dPG) and dPG-based NGs were synthesized as nanoplatforms with the multivalent display of molecules using avidin-biotin interactions. The biotinylated α-CD3 and α-CD28 mAbs were attached to the functional nanoplatform through multivalency avidin-biotin interactions to activate T cells through the T-cell receptor (TCR) in the Jurkat reporter cell line [113]. In anti-cancer therapeutics, the self-homing ability of tumor cells has been exploited as delivery vehicles [131]. Recently, tumor cell membranes are used as a promising and interesting source for packaging nanocarriers due to their excellent tumor cell targeting properties, and also to minimize the non-specific distribution of nanocarriers at non-tumor sites. The cancer cell membrane (CCM)-camouflaged nanoparticles were used early in tumor therapy due to their immune escape and homotypic binding capacities [132]. The fabrication of DOX-loaded dual pH/oxidation-responsive NGs (DNGs) was prepared by inverse miniemulsion using poly(allylamine hydrochloride), formylphenylboronic acid, sodium alginate, and DOX and coated with 4T1 breast cancer cell membranes, which effectively improved their targeting of breast cancer cells. This novel nanoplatform is a promising strategy for tumor-targeted chemotherapy for future translational applications [133].

In cancer therapy, the administration of free anticancer agents is not recommended, as it affects normal cells and causes severe side effects because of systemic biodistribution and high cytotoxicity. Again, the DDS also needs to overcome biological barriers for biodistribution, and target cancer cells, specifically. In this instance, zwitterionic polymers, which have the unique characteristics of biocompatibility, antifouling, long blood circulation time with negligible accelerated blood clearance (ABC) phenomenon, and reduced immune response, are a potential DDS for cancer therapy. Among many, carboxybetaine-based, sulfobetaine-based, and phosphorylcholine-based zwitterionic polymers are widely used in biomedical applications. A novel biodegradable DOX-loaded poly(sulfobetaine methacrylate) (PSBMA) zwitterionic NG for redox-responsive drug delivery was developed against tumors [134]. Lately, a novel hyperthermia responsive poly(2-((2-methacryloyloxy)ethyl) dimethylammonio)acetyl) (phenylsulfonyl) amide (PMEDAPA)-based NG loaded with DOX and modified with glycoprotein transferrin (Tf) exhibited switchable tumor targeting, and DOX release (chemotherapy) regulated by microwave heating (microwave heating therapy) for effective inhibition of human hepatoma cell line (HepG2) bearing tumor xenograft growth [102]. Cancer theranostics requires both imaging and drug delivery systems. Fluorescence imaging by fluorescent probes mainly suffers rapid photobleaching and poor chemical stability, and some metal-based quantum dots are toxic to living organisms. Thus, the development of NGs with intrinsic photoluminescence and high photostability is required. At this juncture, Gyawali et al. [104] developed photo-crosslinkable biodegradable photoluminescent polymers (PBPLPs), incorporated with DOX and surface functionalized with cyclic peptide-based targeting molecule (c(RGDFK) peptide), for tracking intracellular drug delivery to tumor sites in response to pH changes. Thermo-responsive multi-functional NGs synthesized using a PEG-methacrylate-based maleimide-bearing copolymer, crosslinked with a dithiol-based crosslinker, was effectively conjugated with a water-soluble fluorescent moiety (fluorescein–maleimide) and thiol-bearing peptide targeting moiety (cRGDfC peptide) have a strong affinity for integrin receptors of MDA-MB-231 human breast cancer cells for targeted imaging applications [106]. Functionalizable zwitterionic NGs also hold great potential as targeted drug-delivery nanosystems for biomedical applications. Previously, multi-functional biomimetic NGs based on zwitterionic poly(carboxybetaine methacrylate) (pCBMA) were prepared via inverse microemulsion free-radical polymerization with encapsulated dextran, labeled with fluorescein isothiocyanate (FITC-dextran), as a model drug. The presence of abundant carboxylate groups on pCBMA NGs was used to conjugate with cyclo[Arg-Gly-Asp-D-Tyr-Lys] ligands for selective targeted delivery of NGs to primary human umbilical vein endothelium cells (HUVECs) as a model cell system, which expresses α_v_β_3_ or α_v_β_5_ integrins [105,135]. These integrins mediate the interaction of cells with ECM and are involved in tumor cell survival and metastasis and tumor angiogenesis. RGD (Arg-Gly-Asp) peptide is an integrin recognition motif, which can also be used for better cell attachment for different intracellular delivery applications. The RGD-modified cationic NGs obtained by the self-assembly of ethylene diamine and cholesteryl group-modified pullulan (CHP) were used as a nanocarrier of proteins for intracellular delivery into HeLa cells via integrin receptor-mediated endocytosis [107].

Tumor metastasis is the main reason for death among female breast cancer patients. The metastasis of breast cancer cells to lymph nodes, lungs, liver, and bone marrow is inhibited by neutralizing the interaction of cognate ligand CXCL12 (SDF-1) with its overexpressed chemokine receptor CXCR4. Plerixafor (AMD3100), an FDA-approved CXCR4 antagonist, inhibits tumor growth and induces apoptosis and anti-metastasis [136]. NGs are effective nanocarriers for breast antimetastatic cancer chemotherapy in the reducing intracellular environment. Zhang et al. [100] synthesized AMD3100-modified redox-responsive crosslinked dextran NGs with encapsulated DOX (DOX-AMD-DNG) as nanocarriers for targeting CXCR4 receptors of the murine (Balb/C) breast cancer 4T1 cell lines, which exhibited higher cytotoxicity. These NGs also antagonized CXCR4 in U2OS cells expressing functional EGFP-CXCR4 fusion protein, suggesting the inhibition of CXCR4 internalization by NGs as being as effective as free AMD3100 with antimetastatic effects. Even though cancer metastasis is the main challenge in breast cancer therapy, the challenges such as low tumor targetability and slow drug release also need to be addressed. Abraxane^®^, albumin-bound paclitaxel, is the approved drug to treat metastatic breast cancer. For targeting metastatic breast cancer cells, peptides such as LH-RH (luteinizing hormone-releasing hormone), iRGD (9-amino acid cyclic peptide), and tLYP-1 (CGNKRTR) have been used on the nanovehicles with anticancer agents [108]. Many metastatic cells are known to overexpress epidermal growth factor receptor (EGFR) and CD44, which can be targeted by HA and GE11 peptide (YHWYGYTPQNVI), respectively. Interestingly, saporin (saporin-S6), isolated from *Saponaria officinalis* L. seeds, is a type1 ribosome-inactivating protein (RIP) and is used as an immunotoxin in cancer immunotherapy [137]. Chen et al. [108] prepared EGFR and CD44 dual-targeted HA NGs loaded with saporin (Sap-EGFR/CD44-NGs) for enhanced targetability with RIP activity for inhibiting metastatic 4T1 breast cancer in vivo. Cytokine immunotherapy is also an existing promising measure to overcome malignancies that are resistant to conventional therapies. Interleukin-12 is a potent cytokine to induce innate and/or acquired immune responses to eliminate cancer cells and impair neoangiogenesis by inducing IFN-γ-inducible protein-10 (IP-10) [138]. DDS with sustained release of IL-12 was achieved by incorporating recombinant murine IL-12 (rmIL-12) into CHP-based NGs. Repetitive administration of CHP/rmIL-12 induced drastic growth retardation of preestablished subcutaneous fibrosarcoma [139]. Intriguingly, self-assembled NGs of cholesterol-bearing cycloamylose with spermine groups (for superior transfection) (CH-CA-Spe) were used for intra-tumor delivery of siRNA specific to vascular endothelial growth factor (siVEGF) into renal cell carcinoma (RCC) cells through the endocytotic pathway for treating malignancies by suppressing neo-vascularization [101].

Polymer-based nanosystems are extensively used as DDS for cancer therapy and antimicrobial applications. Thus, designing a polymeric DDS for a multitude of biomedical applications needs to be emphasized, and it is achieved by simple switching of polymeric self-assembled structures of spherical micelles to NGs by crosslinking. Previously, Wooley and coworkers prepared crosslinked knedel-like nanoparticles [140,141], and other NG systems were also prepared from crosslinked micelles with pH- and thermo-responsiveness [142]. One of the interesting strategies used to create the NGs from micelles is through UV-initiated thiol–ene coupling (TEC) click chemistry. Very recently, Zhang et al. [93] synthesized polyethylene glycol (PEG)-based amphiphilic ABA-type triblock copolymers and assembled them into micelles, and subsequently crosslinked them using TEC click chemistry to form unimolecular particles of NGs as DDSs for the treatment of cancer and bacterial infections. Short blocks of 5-methyl-5-allyloxycarbonyl-1,3-dioxan-2-one (MAC), an allyl-functional cyclic carbonate, are oligomerized from PEG6k, enabling post-functionalization with 3,4-dihydroxyphenylalanine (dopa)-functional thiols (dopa-thiol), using UV-initiated TEC click chemistry, resulting in amphiphilic ABA-block copolymers with dopa functionalities and allyl groups, allowing for the self-assembly to form micelle, which subsequently forms NGs (100–200 nm) by TEC in the presence of trimethylolpropane tris(3-mercaptopropionate (TMP(SH)_3_) as a crosslinker. The model anticancer drug DOX and antibiotic ciprofloxacin (CIP) were individually loaded into the hydrophobic dopa functional cores of micelles and NGs via physical entrapment, and used as potential DDSs for cancer and antimicrobial therapies.

### 7.2. Functionalized NGs as Nanovaccines

**Figure 4:**
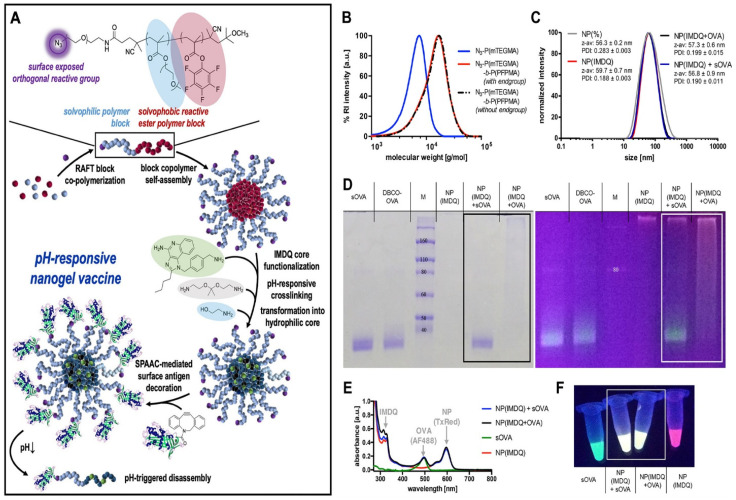
Characterization of TLR7/8-agonist- and protein-conjugated NGs for precise co-delivery of adjuvant and antigen during intravenous antitumor vaccination. (**A**) Synthetic design concept based on double reactive precursor block copolymers that self-assemble into block copolymer micelles with amine-reactive cores and a SPAAC-reactive corona. Using aminolysis of the pentafluorophenyl esters, the cores are covalently functionalized with the TLR 7/8 agonist IMDQ and Texas Red, and then sequentially cross-linked and transformed into pH-responsive NGs. The corona is modified via click ligation of the surface-exposed azides to DBCO-modified (and Alexa Fluor 488-labeled) OVA as a model antigen. (**B**) Size exclusion chromatography of the RAFT-derived reactive homo and block copolymer (before and after removal of the dithiobenzoate end group). (**C**) Dynamic light scattering intensity size distribution plots of the resulting NGs (with and without covalent IMDQ loading), mixed or covalently modified with OVA. (**D**) SDS-PAGE of modified OVA (labeled with Alexa Fluor 488) mixed or covalently conjugated to IMDQ-loaded NGs (labeled with Texas Red) (left, Coomassie staining; right, UV excitation of the fluorescent dyes (red, Texas Red-labeled NG; green, Alex Fluor 488-labeled OVA)). (**E**) UV–vis spectrum of the fluorescently labeled samples and (**F**) corresponding image of the samples upon excitation by a UV lamp. Reproduced from Stickdorn et al. [143] with permission from American Chemical Society, USA.

Nanogel platforms are also used for the development of highly customized and potent nanovaccines (Table 3). The development of effective vaccines against tumors needs the generation of specific humoral and cellular immune responses, especially in the presence of tumor antigen-specific cytotoxic T-lymphocytes (CTLs), to influence the outcome of therapeutic vaccinations. The delivery of antigens for sufficient immunogenicity requires immune-boosting adjuvants in a proper nanocarrier platform with stability. A RAFT polymerization-based NG system with strain-promoted alkyne-azide cycloadditions (SPAAC)-mediated covalent attachment of surface antigen (ovalbumin (OVA), a model antigen (labeled with Alexa Fluor 488) and core functionalization with immune adjuvant (imidazoquinoline-type TLR7/8 agonist) 1-(4-(aminomethyl)-benzyl)-2-butyl-1H-imidazo [4,5-c]quinoline-4-amine (IMDQ) (labeled with Texas Red), which was sequentially crosslinked and transformed into a pH-responsive two-component nanovaccine immunocarrier platform for the safe co-delivery of antigen and adjuvant for intravenous antitumor vaccination. This NG system elicits robust humoral and cellular responses and generates CTL in vitro and in vivo [143,144] (Figure 4). The cancer vaccines need to trigger CD8^+^ T cell activation and anti-cancer CTL responses from major histocompatibility complex (MHC) class I antigen presentation of tumor antigens by antigen presentation cells (APCs) such as dendritic cells (DCs) and macrophages. Vitamin A (retinol), an essential micronutrient, plays a crucial role in both innate and adaptive immunity. Vitamin A, or its active metabolite, retinoic acid (RA), has potency as a vaccine adjuvant for the significant enhancement of antigen-specific antibody production, CD8^+^ effector T cell activation, and mucosal immunity. The amphiphilic pH-sensitive galactosyl dextran-retinal (GDR) NG was constructed for the delivery of encapsulated model antigen OVA (GDR/OVA) as a self-adjuvanted nanovaccine delivery system, in which dextran was conjugated with all-trans-retinal through the hydrazone bond (pH-sensitive linkage), followed by galactosylation, facilitating uptake by DCs. Here, bone marrow-derived dendritic cells (BMDCs) were used as an in vitro cell model to evaluate the effect of GDR on DC maturation, antigen uptake, and MHC I antigen cross-presentation, and the anticancer effect of GDR/OVA was evaluated in a mouse model. The lysosomal rupture, triggered by pH-sensitive nanoparticles in DCs, might be a plausible strategy to elevate intracellular reactive oxygen species (ROS) production, which promotes the MHC class I antigen cross-presentation for improving the efficacy of cancer vaccines [145]. Previously, they reported the use of synthesized bioreducible cationic alginate-poly(ethylenimine) polysaccharide NGs as a novel vaccine delivery system. It also served as a potent vaccine adjuvant to improve vaccine-elicited humoral and cellular immune responses by enhancing vaccine-induced antibody production and MHC class I and II antigen presentation by BMDCs, and anti-cancer CTL response through facilitating lysosome escape and cytosolic release of tumor antigens in mouse DCs [146].

Oral administration of the vaccine is a better and more efficient route to deliver the antigens to mucosal sites due to its noninvasiveness. In this oral route of vaccine administration, the delivery system needs to protect vaccine antigens from degradation. Mannan (Mn)-surface conjugated pH-responsive poly(2-hydroxyethyl methacrylate-*co*-methacrylic acid) P(HEMA-*co*-MAA) NGs with “pathogen-like” properties targeting C-type lectin receptors (CLRs) on APCs were synthesized with entrapped cargo (a model antigen), which protected the antigens at low pH values and released at small intestinal neutral pH values for the activation of APCs. Thus, mannan functionalized NGs are a viable strategy for enhancing antigen delivery to APCs and can be an efficient delivery platform for oral vaccinations [147]. The use of biorthogonal chemistry to chemically crosslink NGs or other hydrogels is emerging as an attractive method to produce novel materials for biomedical applications. Responsive NG systems are interesting and important due to their high stability in a nonresponsive aqueous environment. The synthesis of multi-stimuli-responsive NGs, using a bio-orthogonal and reversible reaction for the spatiotemporally controlled release of encapsulated payloads, was performed. These NGs were functionalized dextran polysaccharide, crosslinked with hydrazone linkages, for pH-responsive, reversible, controlled release of the payloads. Furthermore, the thioketal and disulfide linkages in the crosslinkers integrate the NG network to design NGs responsive to the oxidative and reductive environments, respectively, for the release of the encapsulated payloads. These multi-stimuli-responsive design of NGs target a specific tumoral microenvironment and avoids nonspecific and poor tumor selectivity, which can cause severe side effects and resistance to cancer chemotherapy [150]. Mannose-binding cell surface receptors are another attractive therapeutic and diagnostic target. In another instance, hydrophilic glycosylated NGs (sub-100 nm) were prepared via self-assembly, pH-sensitive core-cross-linking of amphiphilic, but fully hydrophobic, block copolymers (an acetylated glycosylated block and a pentafluorophenyl (PFP) activated ester block), and removal of remaining PFP esters and protecting groups, in one pot. Mannosylated NGs promoted ligand-receptor recognition to mannose receptor CD206, (carbohydrate-binding proteins (lectins) and endocytotic receptor on the surface of dendritic cells (DCs)) in solution and on the cell surface of primary DCs, and were efficiently internalized by DCs under physiological conditions. These NGs have immunological applications involving DCs and macrophage subsets in the interior and surface engineering with vaccine antigens and immune-stimulatory molecules, respectively [148].

Cardiovascular diseases (CVD) are the leading causes of death globally, and hypertension is one of the greatest risk factors for CVD. Vaccination can also be a promising approach to treating and preventing hypertension. For the development of a vaccine for hypertension, endogenous pressor substances such as angiotensin (Ang) I, Ang II, and Ang II type 1 receptor (AT1R) have been used as antigens [151,152]. However, the injectable antihypertensive vaccine is associated with the risk of autoimmune kidney diseases and adverse skin reactions. Thus, a cationic cholesteryl-group bearing pullulan (cCHP) NG was effectively used as an antigenic protein-delivery system for adjuvant-free intranasal vaccines to APCs in the nasal epithelium [153]. Nevertheless, several studies report that hypertension significantly increases pneumonia mortality rates in elderly patients. Thus, developing an intranasal vaccine for hypertension concomitantly targeting pneumonia is a reasonable strategy. Pneumococcal surface protein A (PspA) is a surface protein expressed by *Streptococcus pneumoniae*, which is the most common causative pathogen in community-acquired pneumonia. The development of cCHP NG incorporated with AT1R partial peptide conjugated to PspA (AT1R–PspA) and cyclic diguanylate monophosphate (di-GMP) as the adjuvant was used as a novel nasal vaccine against hypertension and pneumonia [149].

### 7.3. Functionalized NGs as Wound Healing, Antiviral and Antimicrobial Agents

In virus infection, the entry of the virus into host cells is complex and requires transient multivalent interactions with different cell surface receptors. Many human viruses, including herpesviruses, arteriviruses, papillomaviruses, and flaviviruses attach to heparan sulfate (HS) proteoglycans on the human cell surface, triggering a cascade of events resulting in the entry of viruses [154]. Even though antiviral vaccines are available, most RNA viruses continue to mutate and evolve into new variants, making vaccination strategy unavailing. Therefore, antiviral agents as a preventive measure are commonly used against many viral infections. In the development of antiviral agents, it is necessary to block the initial interactions of virus particles with receptors and to shield viral particles from multivalent interactions. Negatively charged functional groups (sulfonate, sulfate, or hydroxyl groups) and functionalized polymeric surfaces have been used as multivalent inhibitors of viral entry. Dendritic polyglycerol sulfate NG particles with different degrees of flexibility with defined sizes (100–200 nm) are robust viral inhibitors. These sulfated NGs block Herpes simplex virus type 1 (HSV-1) attachment to cell membranes by mimicking cellular HS, which multivalently interacts with viral glycoproteins, and thereby blocks entry and infection [155]. Carboxylic acid-terminated polymers (polycarboxylates) are also capable of inhibiting human immunodeficiency virus (HIV) and herpes simplex virus (HSV). The FDA-approved cellulose acetate phthalate (CAP) as nanoparticles has selectivity and antiviral activity against HSV types 1 and 2. CAP in micronized form binds to envelope glycoprotein gp120 of HIV-1 and impairs their infectivity [156]. Some viral infections caused by the influenza A virus (IAV) cause severe illness and mortality worldwide. IAV are pleomorphic (spherical and filamentous) and enter the host cell by multivalent strong binding of its trimeric spike hemagglutinin (HA) proteins to the exposed sialic acid (SA) residues of the glycocalyx on the host cell surface [157]. Inhibition of IAV infection with multivalent sialic acid inhibitors is a promising strategy to prevent virus binding to host cells of the respiratory tract. However, optimal geometry and optimal ligand presentation on multivalent scaffolds are essential for efficient viral inhibition. Bhatia et al. [158] demonstrated that linear and dendritic polyglycerol sialosides (LPGSA and dPGSA) with SA densities of 20–25% for dPGSA and 40–70% for LPGSA were optimum for efficient IAV inhibition. In another instance, medium-sized deformable flexible multivalent NGs containing dendritic and linear polyglycerol sialosides (dPGSAN_3_ and LPGSAN_3_) with 15 and 40% SA residues, respectively, were synthesized and efficiently inhibited the binding of influenza A/X31 (H_3_N_2_) to host cells, and thereby entry. Highly flexible sialyated NGs showed improved IAV inhibition, and the NG prepared by crosslinking of LPGSAN_3_ with dPG-cyclooctyne blocks the adhesion of the virus to host cells (MDCK-II) up to 98.2 ± 1.8%, with IC_50_ values in low picomolar concentrations [159]. The hepatitis B virus (HBV) causes hepatitis B, a potentially life-threatening liver infection that may lead to death from liver cancer and cirrhosis. Delivering antiviral nucleoside analogs to hepatocytes improves therapeutic efficacy and cures hepatitis B and hepatocellular carcinoma (HCC). Lactosaminated-human serum albumin (LHSA) ligand selectively targets asialoglycoprotein receptor (ASGP-R) overexpressed on the parenchymal cell surface of hepatocytes. LHSA-conjugated maleimide-functionalized-poly(lactic-co-glycolic acid (PLGA) loaded with lamivudine nanoparticles (LHSA-Mal-PLGA-Lam NPs) can be used as promising hepatocyte targeted antiretroviral DDS [160].

**Figure 5:**
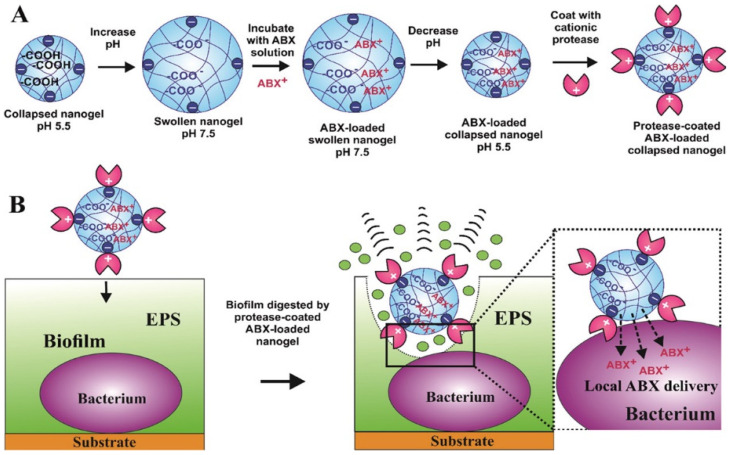
(**A**) The schematic diagram for the loading of the Carbopol Aqua SF1 NG with an antibiotic (ABX^+^) (ABX = ciprofloxacin) followed by surface coating with protease (Alcalase 2.4 L FG). (**B**) Diagram of the mechanism of action of the Carbopol Aqua SF1–Alcalase 2.4 L FG NG particles on biofilms adhered to a substrate. Reproduced from Weldrick et al. [165] with permission from American Chemical Society, USA.

For bacterial infection inhibition, several flexible nanosized functionalized scaffolds, such as the graphene sheets wrapping *E. coli* bacteria, have been employed. Pathogenic bacteria can be phagocytosed by macrophages, which makes them evade the immune system and protects them against antibiotics attack, which makes them ready for recurrent infections [161]. Thus, to prepare any strategy for antibacterial activity, it should improve antibiotic targeting and overcome drug resistance mechanisms with high local drug concentrations with fewer side effects. The development of antibiotic delivery systems with the capacity to efficiently deliver drugs into macrophages improves antibiotic therapy against intracellular infections. Moreover, macrophages can also transport drugs to the site of infection by chemotactic mechanisms [162,163]. Utilizing a mannosyl ligands conjugated PEG shell and polyphosphoester core-crosslinked NG as the drug nanocarrier, the hydrophilic antibiotic drug (vancomycin) was target delivered to macrophages to treat bacterial infections by the active phosphatase and/or phospholipase enzymatic degradation by bacterial enzymes. These NGs provide macrophage targeting and lesion site-activatable drug release properties along with enhanced bacterial growth inhibition [164]. Even novel nanotechnology was used to produce anti-biofilms NGs. A protease-functionalized NG of antibiotics carrier was used to disrupt the extracellular polymeric substance (EPS) matrix of biofilms to exhibit antibiofilm activity. Serine endopeptidase/endoprotease Alcalase 2.4 L FG-coated Carbopol Aqua SF1 NGs with encapsulated cationic ciprofloxacin was used against wound-associated biofilm-forming bacteria, *Staphylococcus aureus*, *Pseudomonas aeruginosa*, *S. epidermidis*, *Klebsiella pneumoniae*, *Escherichia coli*, and *Enterococcus faecalis*, to decrease biofilm mass and produce a substantial reduction in bacterial cell density [165] (Figure 5). Some functional aqueous NGs can also be potentially used to prevent microbial infections as well as to promote tissue regeneration. This competition between bacterial adhesion and cellular tissue integration on the same biomaterial surface is phrased as a “race for the surface” [166]. Kather et al. [167] devised a new strategy in the development of NG-based functional coatings for dental implants with better tissue integration of human gingival fibroblasts and long-term safety from oral pathogens. These NGs, with functional and hydrophilic PEG decorated with a controlled amount of surface-drafted and covalently attached eugenol (IE), an antiseptic and antibacterial bioactive compound from clove spice, allow the growth of gingival fibroblasts. Similarly, these NGs differing in spacer length, and the amount of isoeugenol (IE) functionalization were tested against peri-implantitis-associated obligate and facultative anaerobic bacterial species, and the antimicrobial effect was dependent on the spacer chain length, particle size, and IE concentration. NG9-3 and NG9-4 had inhibitory effects on all Gram-positive species and *Porphyromonas gingivalis* and *Prevotella intermedia* [168].

For wound healing, the wound needs treatment with a nanocarrier that can improve wound healing and control antimicrobial infection. NGs can also involve in the controlled/sustainable release of active substances such as growth factors, cytokines, or morphogenetic factors, and are also involved in regulating cell behavior by fine-tuning the texture and mechanical properties of the scaffold [169]. These NGs, with active substances are used in wound healing. Chitosan (CS)-based NGs were prepared by an ionic gelation method with pentabasic sodium triphosphate (TPP), and interleukin-2 (IL-2)-loaded chitosan-TPP NGs, stimulated the release of interferon-gamma (INF-γ) and lymphocytes for wound healing, and they also decreased the malondialdehyde (MDA) levels and increased the glutathione (GSH) levels of wounded tissues in rats [170]. The development of hybrid hydrogels with rapid hemostasis and sustainable antibacterial property was reported [171]. These hybrid hydrogels were composed of aminoethyl methacrylate hyaluronic acid (HA-AEMA), and methacrylated methoxy polyethylene glycol (mPEG-MA) loaded with CLNs (abbreviated as Gel@CLN). The chlorhexidine diacetate-loaded NGs (CLNs) were prepared by the enzymatic degradation of CHX-loaded lysine-based NGs. These Gel@CLN hybrid hydrogels showed a prolonged release period of CHX over 240 h with antibacterial properties, and rapid hemostasis capacity with accelerated wound healing ability for wound dressing applications [171]. A novel nanocarrier, based on lightly cross-linked and highly hydrophilic acrylate copolymer NG particles (Carbopol Aqua SF1), was loaded with antimicrobial hexametaphosphate salt of chlorhexidine (CHX) (CLC), followed by surface functionalization with cationic polyelectrolyte poly(diallyldimethylammonium chloride) (PDAC), to form PDAC-functionalized CLC. These PDAC-functionalized CLC NGs showed antimicrobial, antifungal, and antialgal activities on *E. coli* and *S. aureus*, *Saccharomyces cerevisiae* and *Chlamydomonas reinhardtii*, respectively [172]. Similarly, in another instance, the antimicrobial compound berberine was encapsulated on a PDAC surface functionalized on polyacrylic acid (PAAc)-based NGs. These cationic NG carriers exhibited enhanced antimicrobial activity on *E. coli* and *C. reinhardtii*. These cationic functionalized NG-based approaches can be used to develop more effective wound dressing, disinfecting agents, antimicrobial surfaces, and antifouling formulations [173]. Even the mild pyrolysis of a mixture of dextran 70 (DEX) and the crosslinker 1,8-diaminooctane (DAO) at 180 °C/3 h formed multi-functional carbonized NGs (DAO/DEX-CNGs) with NG-like structures and functional groups from their precursor molecules. These CNGs manifest broad-spectrum antibacterial activity against marine bacteria *Vibrio parahaemolyticus*, the causative agent for acute hepatopancreatic necrosis disease (AHPND), and MDR strains by disruption of bacterial membranes, producing oxidative stress and neutralizing PirAB toxins [174]. By changing the surface functionalization chemical groups, it is possible to tune and control the drug release in DDS. The synthesized poly(maltose) (p(MAL)) particles with divinyl sulfone (DVS) as crosslinkers via microemulsion technique demonstrated versatility in the drug release of sodium diclofenac by modifying the surface with ethylene diamine (EDA), polyethylenimine (PEI) and taurine (TA). These p(MAL) nanoparticles exhibited antibacterial activity against *E. coli* and *S. aureus* upon chemical modification with PEI [175] (Table 4).

### 7.4. Functionalized NGs for Tissue Engineering and Regenerative Medicine

NGs with functionalizations are also utilized in tissue engineering scaffolds for the controlled release of bioactive molecules (growth factors, cytokines, genes, chemokines, etc.) for cell proliferation and differentiations, and to control the properties of the scaffolds (Table 5). Biocompatible synthetic polymers can also be employed to create well-defined microenvironments with controlled physicochemical properties for cell culture and tissue engineering. The structural material with chemical modifications of biological or chemical ligands is required for cell adhesion and differentiation for tissue engineering and regenerative medicine. A biomaterial design that employs functional alpha-cyclodextrin (α-CD) nanobeads with chemical (hydrophobic, hydrophilic, or charged groups) and/or biological (cell integrin-binding peptides; YRGDS) functionalities, threaded onto linear acrylated end-functionalized PEG polymer necklaces to form multi-functional hydrogels, was used for cell culturing and differentiation to tissue engineering and regenerative applications [176]. Aptamer-functionalized hydrogels can be used for the controlled release of proteins and oligonucleotides for regenerative medicine. The strategy of controlled release by using DNA oligonucleotides as affinity ligands to hybridize, capture and release therapeutic oligonucleotides through enzymatic and physical triggers, was performed. The functionalization of polyacrylamide gel with anti-platelet-derived growth factor-BB (PDGF-BB) DNA aptamer as a model system containing PDGF-BB was developed for the sustained-release kinetics of PDGF-BB [177]. The 3D conformation of DNA aptamers recognizes their target with high specificity through various non-covalent interactions.

NGs formed by the ion pairing between polyanions and polycations to form polyelectrolyte complexes (PECs) is a spontaneous process formed by simple mixing in aqueous media. PEC-based NGs from polysaccharides bring great attention to DDS due to their easy preparation and biocompatibility. Van Le et al. [186] was the first to prepare PEC-NGs from thermoresponsive polysaccharide derivatives for DDS. The PEC-NGs were prepared using polyanionic hyaluronic acid (HA) or HA-M2005 (HA functionalized with diblock copolyether monoamine, Jeffamine^®^ M-2005, with LCST temperature of 14–30 °C), which electrostatically complexed with polycations diethylaminoethyl dextran (DEAE-D) or poly-L-lysine (PLL), along with the encapsulated curcumin as a model anti-inflammatory drug for delivery. Intriguingly, Nita et al. [187] developed an intelligent biomaterial of multi-responsive NGs by crosslinking poly (itaconic anhydride-co-3,9-divinyl-2,4,8,10-tetraoxaspiro [5,5] undecane) (PITAU) copolymers with different molar ratios between comonomers with 1,12-dodecandiol, and modifications of itaconic anhydride moieties in the copolymer yielded new structures. Depending on the behavior of the PITAU copolymers, a network with increased functionality was achieved to couple bioactive compounds and also present dual pH and temperature-sensitive characteristics. These NGs were also tested as DDS using anti-inflammatory drug diclofenac as a model drug.

Neo-vascularization is an important process in the regenerative medicine and wound healing process. It is also accompanied by the proliferation of host cells and the chemotactic attraction of circulating endothelial progenitor cells (EPCs) to the wound site. The differentiation of EPCs into endothelial cells (EC) needs a change in the micro-environmental conditions by introducing genes or drugs into the cells. Previously, synthetic polymers based on Pluronic F-127 have been used as vehicles for the delivery of these required components due to their nontoxicity [188]. Glycosylated vascular endothelial growth factor (VEGF_165_) is one of the several forms of VEGF found in the blood vessels in a biologically active form. Basic fibroblast growth factor (bFGF) also involves in neovascularization by stimulating vascular EC mitogenesis for wound healing and pathologic angiogenesis. Yang et al. [183] prepared negatively charged heparin-modified supramolecular pluronic NGs with encapsulated bFGF and complexing VEGF_165_ pDNA on the pre-coated positively charged polyethylenimine (PEI) (HP–bFGF/PEI) as a gene delivery vehicle to EPCs to promote EC differentiation and neovascularization in an ischemic limb model system.

**Figure 6:**
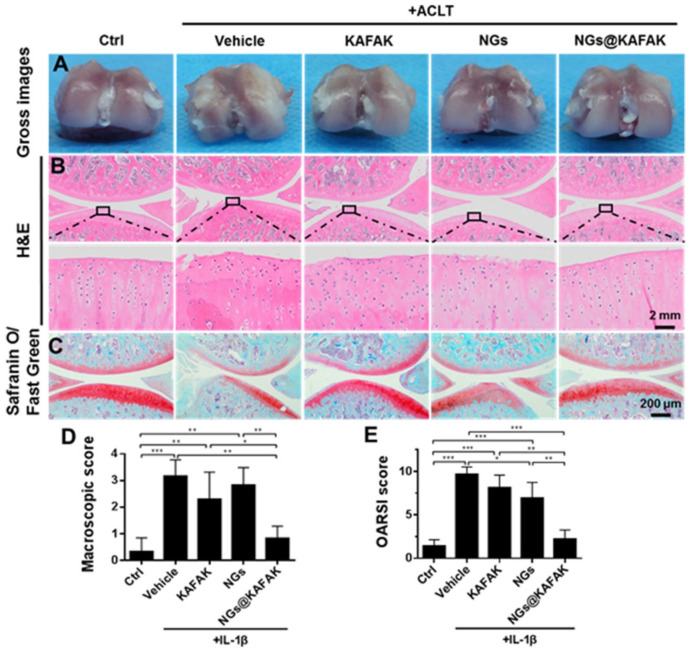
Intra-articular injection of NGs slows down cartilage degeneration in anterior cruciate ligament transection osteoarthritis (ACLT) induced rat osteoarthritis model. (**A**) Macroscopic appearance of cartilage from tibial plateaus, (**B**) H&E staining of knee joints, (**C**) Safranin O/Fast Green stained sections of knee joints, (**D**) The macroscopic observation scores of knee joints, (**E**) Cartilage degeneration evaluated with the Osteoarthritis Research Society International (OARSI) scoring system. (* *p* < 0.05, ** *p* < 0.01 and *** *p* < 0.001). Reproduced from Li et al. [180] with permission from Elsevier.

Osteoarthritis (OA) is a degenerative and heterogenous joint disease by inflammatory and metabolic factors, causing loss of cartilage, synovial inflammation, and alterations in subchondral bone [189]. The inflammation of joint components, notably the articular cartilage, is the pathogenesis of OA altering the ECM composition and organization. The activation of inflammation-induced and stress-induced signaling pathways leads to the secretion of pro-inflammatory cytokines such as IL-1β and TNF-α involved in cartilage degradation [190]. The treatment of OA requires the delivery of bioactive molecules such as vasoactive peptides (endothelin-1 and bradykinin) into the joints to limit inflammation and reduce cartilage degradation. The functionalization of chitosan with type A endothelin receptor antagonist (BQ-123-CHI) and/or hyaluronic acid functionalized with a type B1 bradykinin receptor antagonist (R-954-HA) decreased inflammatory and catabolic markers and protected cartilage in an OA equine cartilage organoid model [178]. The cartilage degeneration releases proteoglycan-degraded products and damage-associated molecular patterns (DAMPs), which causes the activation of immune cells in the synovium to secrete pro-inflammatory cytokines such as IL-1, IL-6, and TNF-α [191]. The covalent attachment of a peptide that inhibits a kinase involved in inflammation, mitogen-activated protein kinase-activated protein kinase 2 (MAPKAP2 or MK2) with cell-penetrating peptide was used as a method of delivering biologically active peptide [192]. Cell-penetrating peptide, KAFAKLAARLYR, is based on anti-thrombin III heparin-binding domains, which was conjugated with anti-inflammatory cationic MK2 inhibiting peptide KALARQLGVAA, a therapeutic domain (TD), forming KAFAKLAARLYRKALARQLGVAA (abbreviated as KAFAK) for the inhibition of MK2 and decreased the expression of IL-6 and TNF-α. The KAFAK-loaded PEGylated thermosensitive poly(N-isopropylacrylamide) (pNIPAM) nanoparticles with degradable disulfide crosslinking (NGPEGSS) were used to reduce ex vivo inflammation in chondrocytes [179]. However, KAFAK has low enzymatic degradation stability, and rapid clearance by the lymphatic system, with a short half-life and low bioavailability [193]. To make long-circulating nanocarriers for intra-articular (IA) delivery of KAFAK, to improve drug residence time and biodistribution in the joint, nanoparticles consisting of anionic fucose-rich sulfated polysaccharide, fucoidan (Fu), and cationic glycol chitosan (GC) with KAFAK were prepared via electrostatic interaction based on the positive charge of KAFAK, with further crosslinking with genipin to synthesize IA injectable GC/Fu@KAFAK NGs [35,180] (Figure 6). For the local application of drugs or biomolecules, including cytokines and growth factors in damaged tissue for regenerative medicine, NG-based hydrogel composites can be used. This approach of using natural or synthetic biomaterial as artificial scaffolds with the bioactive component for sustained release helps in tissue repair of tissue defects. For instance, bone-inducing growth factor bone morphogenetic protein 2 (BMP-2) is required for repairing critical-sized bone defects and in the reconnection of the tendon to bone [194]. The sustained release of BMP-2 at the target site is required for increased efficacy and to avoid ectopic bone growth and soft tissue inflammation caused by the fast release of high doses. To restore the tissue transition between bone and tendon, an artificial scaffold with growth factors that can exert a trigger on cell recruitment and proliferation is needed. Polycaprolactone (PCL) fiber mats with different hydrophilic surface modifications were functionalized with a chitosan NG coating containing BMP-2 for sustained release without any loss of bioactivity in vitro [195].

Hydrogels functionalized with glycomimetic peptides of naturally occurring carbohydrates–polysialic acid (PSA) and human natural killer cell epitope (HNK-1) are shown to encourage nerve regeneration and axonal targeting [196]. Astrogliosis is the process by which astrocytes respond to central nervous system (CNS) damage and disease with a variety of potential changes in gene expression, cellular structure, and function. Activated astrocytes are those astrocytes’ responses to injury or disease [197]. Astrocytes in the A1 state have a pro-inflammatory effect, affecting the neuronal cells, whereas the A2 state can upregulate neurotrophic factors involved in neuronal regeneration. Activation of astrocytes for neuronal regeneration can be achieved by specific pharmacological or biological therapy through different nanosystems. However, these approaches are hindered in neurons by the microglia/macrophage’s phagocytic mechanisms. Designing a nanovector system with biocompatible and drug-loading and release capacity targeting activated astrocytes is important. NH_2_-Cy5 functionalized NGs were the most promising for targeting astrocytes, which are biocompatible and reduce microglia/macrophage recognition and uptake. NGs with conventional PEG surface coating or recombinant CD47 protein limit the phagocytic clearance by macrophages [198]. The internalization of Rolipram-loaded NH_2_-Cy5 functionalized NGs by astrocytes was mediated by a clathrin-dependent endocytic pathway and underwent lysosomal degradation for the release of anti-inflammatory drug Rolipram, which inhibits NF-κB translocation into the nucleus and reduces transcription of pro-inflammatory genes in an in vivo spinal cord injury (SCI) model for astrogliosis [182]. The nucleus pulposus is a soft gelatinous portion present in the center of the intervertebral disk (IVD) and absorbs compressive pressure, allowing disk mobility. It consists of a water-rich ECM with chondrocyte-like nucleus pulposus cells [199]. For the regeneration of the nucleus pulposus tissue, the cells must produce an appropriate proteoglycan-rich matrix, as this is essential for the functioning of the intervertebral disk [200]. An injectable, laminin-111 functionalized poly(ethylene glycol) (PEG-LM111) biomaterial was developed for nucleus pulposus cell delivery to IVD [181]. Mufamadi et al. [201] also developed a nanocomposite hydrogel system with the embedded FITC-labeled functionalized nanolipobubbles within the crosslinked hydrogel network. These nanolipobubble surfaces engineered with synthetic peptide (S1) provide effective intracellular delivery of drugs into PC12 neuronal cells via specificity for over-expressed surface receptors for the management of neurodegenerative disorders.

Alzheimer’s disease (AD) is another neurodegenerative disease, which is closely correlated with the dysfunction of insulin, insulin receptors (IR), and IR signaling. The administration of insulin could be another interesting strategy for AD treatment. An insulin (In)-conjugated NG (NG-In) system was used as a suitable carrier for insulin delivery in the brain for AD therapy [202]. A carboxyl-functionalized poly(N-vinyl pyrrolidone) (PVP) NG produced by ionizing radiation was covalently attached to insulin (NG-In), triggering insulin signaling via AKT activation by binding to the IR. NG-In as a nasal spray was efficiently transported across the blood–brain barrier and exhibited neuroprotection against dysfunction induced by amyloid β (Aβ), which is involved in AD [184]. Cationic polymers such as polyethylenimine (PEI) can also be used as a vehicle to deliver genes to stem cells and somatic cells. The shielding of PEI with anionic polymers of hyaluronic acid (HA) can effectively deliver genes into human mesenchymal stem cells (hMSCs). HA interacts with the CD44 glycoprotein receptor on the plasma membrane of hMSCs, and facilitates receptor-mediated internalization of HA-functionalized PEI/pDNA NGs to deliver genes into stem cells for regenerative medicine [185].

### 7.5. Miscellaneous Applications of Functionalized NGs

Novel functionalized NGs as nanocarriers of drugs or other biomolecules or imaging moieties for a site-specific or time-controlled delivery can be used for various other biomedical applications depending on the characteristics of the encapsulated or loaded payload and the target (Table 6). Among different polymeric nanocarriers, NGs for non-irritating delivery vehicles to increase the dermal bioavailability of therapeutics are a promising improvement. Skin is a complex biological barrier to protect underlying tissues from pathogenic entry, environmental irritants (chemical, biological and mechanical), and dehydration. The amphipathic stratum corneum (SC) is the outermost layer of the epidermis, and is mainly involved in restricting permeability [203]. A rational design of NGs with polymeric functionality to tailor their properties is essential to overcome the biological barrier of the skin by effectively disrupting the ordered SC and increasing the drug permeation. Molecules including alcohols, fatty acids, pyrrolidones, phospholipids, and sulfoxides have been used as chemical penetration enhancers. Among these chemical enhancers, dimethyl sulfoxide (DMSO) is one of the most potent and effective penetration enhancers [204]. These tailor-made trans-dermal delivery vehicles were developed by covalently functionalizing the NGs with such methyl sulfoxide side groups. Amphiphilic sulfoxide-based NGs (NG-SoMe) are superior in interaction with SC with increased topical delivery efficacy of Nile red (NR) to the viable epidermis (VE) of excised human skin [205].

Targeted nanomedicine of inhalation therapy using RNA interference (RNAi) of specific gene silencing of target genes has the potential for the treatment of different respiratory pathologies, outperforming other conventional therapies. To perform nucleic acid delivery, siRNA-targeted cellular delivery to pulmonary cells needs to be achieved. The use of membrane-associated surfactant protein B (SP-B) in the functional pulmonary surfactant (PS) can assist in the cytosolic delivery of payloads through endocytosis in the pulmonary cells. Merckx et al. [215] demonstrated the potential of SP-B in enhancing the cellular siRNA delivery of proteolipid-coated NGs to alveolar macrophages in the treatment of inflammatory pulmonary pathologies for inhalation therapy. Curosurf^®^ (poractant alfa) was clinically used as PS. Curosurf^®^-coated siRNA-loaded dextran NGs (siNGs), as a nanocarrier was lyophilized as a dry powder without cryo- or lyoprotectants, for reconstitution without affecting their physicochemical properties for the cytosolic delivery of siRNA into lung epithelial cell lines through nebulization via a vibration mesh nebulizer for inhalation therapy [206,216]. A versatile multi-functional amphiphilic NG with pH- and reduction-sensitive internal networks with reactive hydrophilic shells for surface modification by orthogonal functionalization of the interior (core) using active ester chemistry and surface (shell) using CuAAC reaction, which further enables sequential modifications of the reactive precursors. For the interior, acidic 4-aminomethylphenylacetic acid (AMPAA) or basic histamine (HIS) functionalities were used in combination with hydrophobic groups to prepare pH-sensitive amphiphilic NGs, while the hydrophilic shell was functionalized with an azide-containing model compound (rhodamine). Thus, the orthogonal functionalization of the core and shell demonstrates the potential to develop highly functional nanocarriers with tailored properties for advanced applications [217].

Autonomously reporting sensor materials are another interesting class of smart materials, which are used in the robust, sensitive, and selective detection of bacterial enzymes/bacteria [218]. In the rationale of fabricating hydrogel nanomaterial-based autonomously reporting sensor materials, an engineered strategy of anodic aluminum oxide (AAO)-mediated nanoporous extrusion was used to fabricate high-surface-area NGs/nanofibers conjugated to a colorimetric indicator for the detection of bacterial enzymes. The ketone-functionalized pullulan derivative was used as the precursor for the preparation of chemically crosslinked NG fibers. The functionalization of pullulan acetoacetate (PUAA) derivatives with fluorogenic substrate 4-methylumbelliferyl-β-D-glucuronide hydrate (MUGlcU) (PUAA-MuGlcU) was enzymatically hydrolyzed by β-glucuronidase-producing *Escherichia coli* by cleaving β-glycosidic linkage in the PUYAA-MUGlcU NG fibers, and fluoresces by the release of 4-MU, which indicates the presence of specific bacteria and/or bacterial enzymes [207].

Even nanosystems are used for the transportation of oxygen using hemoglobin-based nanocarriers, which have tremendous applications in organ transplantation, blood transfusions, hemorrhages, and anti-tumor therapy. Hemoglobin (Hb) is one of the most important functional proteins in living systems, which is responsible for the transport of oxygen, carbon dioxide, and other gases. The delivery systems such as vesicles, micelles, nanoparticles, and hydrogels of various sizes are used for Hb delivery. In 2014, Wang et al. [209] prepared a novel thermoresponsive hemoglobin-polymer conjugate between the lysine amino groups of Hb and the carboxyl groups of copolymer poly(N-isopropylacrylamide) grafted carboxylated dextran (HOOC-Dex-*g*-PNIPAAm) by single electron transfer living radical polymerization (SET-LRP) and post-carboxylation. Upon heating above the LCST, the conjugate forms isolated uniform spherical nanoparticles with relatively low critical micelle concentration (CMC). The redox activity and gas binding capacity of the Hb conjugate showed Hb bioactivity after conjugation. Using hydrophilic 3D NGs with good biocompatibility, stability, and functional groups, has several advantages as a nanocarrier for protein delivery. Dextran-*g*-succinic anhydride-*g*-dopamine conjugate (Dex-SA-DA) was prepared by open-ring and amidation reactions and self-assembled into NGs by simple pH adjustment. The in situ cross-linking and aldehyde-functionalization of these NGs by NaIO_4_ oxidation in water, and the post-conjugation of Hb to these swollen functionalized NGs, form Hb-loaded NGs (HbNGs) by Schiff base reaction under mild conditions, which exhibits high stability, oxygen affinity and hemocompatibility for these oxygen carriers [208].

Enzymatic biocatalytic reactions are greatly used in the production of various active pharmaceutical ingredients (APIs) through sustainable green chemical processes. Among various enzymes, lipases are considered one of the most important industrial biocatalysts in several chemo-, regio- and enantio-selective transformations [219]. However, lipase activity is highly susceptible to organic solvents, high temperatures, and extreme pH, limiting their applications. Chemical modifications and immobilizations are well-known methods used to protect the lipase active center and prevent it from denaturation. The use of ionic liquid to modify the histidine of porcine pancreatic lipase (PPL) by chemical modification increased the hydrolytic activity of PPL more than natural lipase [220]. The chemical modification of lipase from *Candida rugosa* (mCRL) was performed by introducing ionic liquids with vinyl functional groups on the surface of CRL to prepare lipase NGs for in situ polymerization in aqueous phase, yielding lipase molecule as the core and the polymer layer as the shell. This enhanced the catalytic activity of mCRL to 1.2 times higher than that of the natural CRL, and also showed improved pH and thermal stability and tolerance to organic solvents. Such functionalized ionic liquid-modified enzyme NGs can be a potential biocatalyst for industrial applications [221]. Similarly, novel immobilized *Candida rugosa* lipase NG was developed by the modification of ionic liquid with the vinyl functional group, and was used as an excellent biocatalyst in the enzymatic synthesis of vitamin E succinate based on the principle of non-aqueous enzymology [211].

Detection of glucose, particularly in diabetic patients, is very important to combat other associated diseases. The development of an efficient glucose detection system is a paramount measure to control the most appalling complications. The colorimetric detection of glucose is one of the most common methods due to its simplicity, low cost, efficiency, and applicability. Colorimetric detection is based on the detection of hydrogen peroxide (H_2_O_2_) by the colored product after enzymatic reactions. Generally, glucose is oxidized by the enzyme glucose oxidase (GOX) into gluconic acid and H_2_O_2_. The produced H_2_O_2_ oxidizes a chromogenic agent in the presence of enzyme peroxidase to form a colored product. The immobilization of GOX and peroxidase in proximity to each other can bring the detection procedure as a single-step method. Cheon et al. [222] prepared iron oxide (Fe_3_O_4_) magnetic nanoparticles (MNPs)-embedded GOX-copper hybrid nanoflowers (MNPs-GOX NFs), which exhibited peroxidase-mimicking activity as well as substrate channeling for the detection of glucose. Another composite prepared using 2D material graphdiyne (GDY) with immobilized ferrous ion and GOX was used in one-step blood glucose detection [223]. Very recently, Fan et al. [210] fabricated GOX-hemin NG (GHN) via polymerization on the surface of GOX, where hemin was immobilized on GOX with peroxidase activity together with GOX, forming a highly sensitive one-step glucose detection system via a colorimetric assay at room temperature with good thermostability and organic solvent resistance ability. Earlier, aptamer-functionalized polyacrylamide hydrogel microparticles (10–50 µm) were used for ultrasensitive visual detection of mercury(II) (10 nM) and adenosine [213].

**Figure 7:**
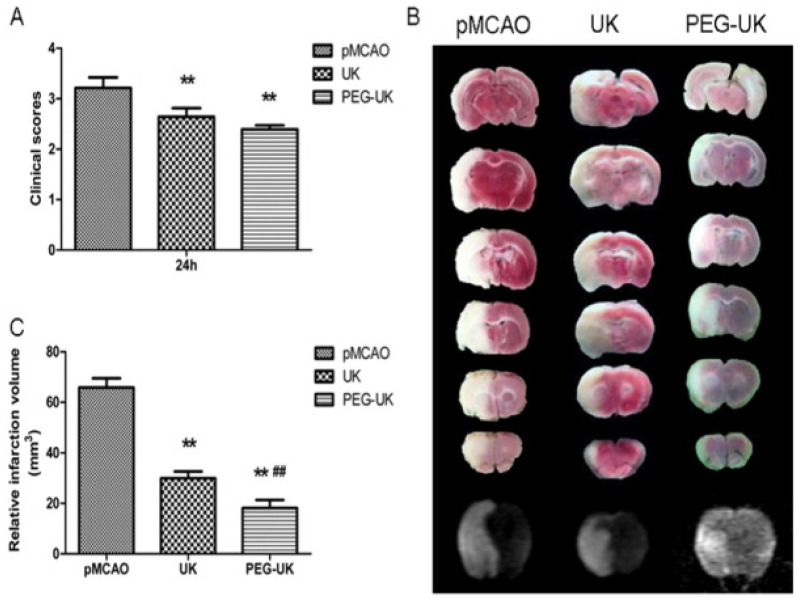
Both urokinase (UK) and PEG-UK reduced neurological deficits. (**A**) The clinical scores. (**B**) Representative images of the threshold of toxicological concern (TTC) straining and diffusion-weighted imaging (DWI) in each group: the infarcted part stained white, whereas the normal part-stained red. (**C**) The mean relative infarction volume of each group. ** *p* < 0.01 vs. pMCAO; ## *p* < 0.01 vs. UK. Reproduced from Cui et al. [214] with permission from Elsevier.

In another biomedical application, NGs can also be used as metal chelators in the chelation therapy of heavy metals [224]. In particular, iron-chelation therapy is an effective way to treat iron overload, where elevated levels of iron in the blood play important roles in the disease progression of Alzheimer’s and Parkinson’s diseases and cancer angiogenesis. Liu et al. [212] designed and synthesized oxidation-sensitive iron chelating NGs (oxNG–DFO) using free radical polymerization of acrylamide (AAm), oxidation-sensitive host–guest crosslinkers (CL) between β-cyclodextrin (β-CD) and ferrocene (Fc), and iron chelating moieties of deferoxamine (DFO) in reverse emulsion reaction chambers. Under oxidative stress, these nanomaterials can become degraded into smaller chelating fragments and the conjugated DFO reduced the cytotoxicity of the free DFO in the J774A.1 macrophage cell. Similar metal chelators such as ethylenediaminetetraacetic acid (EDTA) and clioquinol derivatives can also be incorporated in the NG for chelating various metals to expand the applications of chelation therapy. In the treatment of stroke, which is another important cause of death, thrombolysis is the most effective therapy. The selective thrombolysis of microcirculatory clots, where the low pH value of the ischemic tissue can be used to induce the selective release of thrombolytic agents [225]. Along with recombinant tissue plasminogen activator (rt-PA), urokinase (UK) is also used as a thrombolytic agent for the treatment of acute ischemic stroke. Cui et al. [214] synthesized pH-sensitive PEG-conjugated urokinase NGs (PEG-UKs). Both free UK and PEG-UK were applied through intravenous tail injection to a rat model of permanent middle cerebral artery occlusion (pMCAO) to investigate their effects by targeting low pH regions of microcirculation. The results showed that the treatment with PEG-UK improved ischemic brain tissue and protected the blood-brain barrier by inhibiting apoptosis and decreasing neurotoxicity in ischemic stroke (Figure 7).

## 8. NGs in Clinical Translation

Many parameters affect the efficacy of NGs in clinical translation. The distribution of NGs in biological tissues is governed by their size, shape, surface properties, and composition. NG size and shape are important in determining the circulation half-life. Spherical NGs have a shorter circulation half-life than rod-shaped particles. The adsorption of serum proteins on their surface also enables opsonization, resulting in clearance, and the neutral surface charge of NGs allows for longer circulation times [21]. Moreover, the NG-delivery system should be compositionally designed according to the application, site of delivery/action, and the nature of payload(s). A myriad of NGs has been synthesized and reported for diverse applications in various biomedical fields. However, there are only a few NG-based systems that have been successfully cleared out in clinical trials, particularly in cancer therapy [5]. Opaxio^TM^ (paclitaxel poliglumex; CT-2103) (formerly known as Xyotax), a polymeric nanodrug formulation, is a covalent linkage of paclitaxel (PTX) to biodegradable poly-L-glutamic acid (PG) (PG-TXL). Currently, it is in clinical trials in ovarian cancer and non-small cell lung cancer (NSCLC) patients. Phase III studies demonstrated greater antitumor efficacy and reduced the side effects of CT-2103; however, there was no significant improvement in the survival of the advanced NSCLC patients [226]. Kitano et al. [227] developed an antigen delivery system comprising a truncated HER2 protein 1–146 (146HER2) complexed with cholesteryl pullulan (CHP) NG. The subcutaneous vaccination of HER2-expressing cancer patients in a phase I clinical study showed HER2-specific CD8+ and/or CD4+ T-cell immune responses, along with the adverse effect of grade 1 transient skin reaction at the site of vaccination. In another instance, CHP with NY-ESO-1 protein complex (CHP-NY-ESO-1) was used as a therapeutic vaccine to immunize esophageal, malignant melanoma, and prostate cancer patients to induce NY-ESO-1 antibody in a phase I clinical trial, which elicited antigen-specific CD4+ and CD8+ T cell immune responses [228]. The dose-escalating clinical trial with CHP-NY-ESO-1 complex vaccine (Drug code: IMF-001) for esophageal cancer patients confirmed the induction of efficient immune responses at 200 µg of complex vaccine [229]. Later, the CHP delivery system was used to deliver the melanoma-associated antigen 4 protein (MAGE-A4). A phase I clinical trial of the CHP-MAGE-A4 vaccine containing 300 µg MAGE-A4 elicited CD8 T-cell and humoral responses when administered subcutaneously in patients with advanced esophageal, stomach, or lung cancer [230].

## 9. Conclusions and Future Perspectives

Nanogels constructed with intelligent polymers are highly responsive toward endogenous and exogenous stimuli such as pH, light, temperature, and so on. The versatility of NGs lies in their multi-functionalities with the modification or functionalization of NGs that improves the features such as stability, biodegradability, biocompatibility, targetability, stimuli-responsiveness, and controlled/sustained release kinetics. The promising novel developments in the fabrication of NGs with tunable mechanical properties and stimuli-responsiveness widen their prospects for controlled/sustained drug delivery systems and imaging for the treatment of various diseases and disorders. Thermoresponsive biosensors can be developed using smart polymeric brush coatings. The temperature-induced changes in the physical state of polymers, based on LCST and glass transition temperature (Tg), can be utilized in the fabrication of NGs for biomedical applications [231]. Furthermore, the elucidation of the role of hydration in the interaction of biomolecules with biomaterials, and the knowledge about interfacial water, can be useful for the design and development of advanced biomaterials for biomedical applications [232]. The investigations on the novel surface engineering modifications and conjugations with unique targeting molecules to desired cell types and intracellular compartments can bring more specificity on activity and avoid adverse side effects. Despite these developments of novel NGs with various functionalities, a substantial number of issues related to their biological interactions, metabolism, pharmacodynamics, and pharmacokinetics also need to be completely addressed with clinical experiments for the safe translation of these NGs from bench to bedside. Moreover, the preparation of smart NGs using biocompatible and naturally available polymers possessing smart/intelligent functionalities and tunable multi-functionalities is regarded to be primitive, and requires considerable progress for widespread applications in theranostic and other biomedical applications.

## Figures and Tables

**Table 1:** FDA-approved commercial polymeric-based nanocarriers.

Commercial Name	Chemotherapeutic Moiety	Company	Applications
Abraxane^®^ (paclitaxel protein-bound particles for injection suspension) (albumin-bound)	Paclitaxel	Abraxis Bioscience, Los Angeles, CA, USA	Breast cancer, metastatic breast cancer, metastatic non-small cell lung cancer (in combination with carboplatin), metastatic adenocarcinoma of the pancreas (in combination with gemcitabine)
ADYNOVATE^®^ (antihemophilic factor (recombinant))	Factor VIII or Antihemophilic factor (Recombinant)	Takeda Pharmaceuticals, Lexington, MA, USA	Haemophilia A
Cimiza^®^ (certolizumab pegol) injection	Tumor necrosis factor (TNF) blocker	UCB, Inc, Smyrna, GA, USA	Crohn’s disease, Rheumatoid arthritis, Psoriatic arthritis, ankylosing spondylitis, non-radiographic axial spondyloarthritis, plaque psoriasis
Copaxone^®^ (glatiramer acetate injection)	Glatiramer acetate	Teva Neuroscience, Kansas City, MO, USA	Multiple sclerosis
Eligard^®^ (Leuprolide acetate) for injectable suspension	Luteinizing hormone-releasing hormone (LHRH) agonists	Tolmar Pharmaceuticals Inc., Fort Collins, CO, USA	Prostate cancer
Neulasta^®^ (pegfilgrastim) injection	Leukocyte growth factor	Amgen Inc., Thousand Oaks, CA, USA	Neutropenia, Hematopoietic subsyndrome of acute radiation syndrome
Oncaspar^®^ (pegaspargase)	L-asparaginase	Servier Pharmaceuticals, Boston, MA, USA	Acute lymphoblastic leukemia
PegIntron^®^ (peginterferon alfa-2b) (subcutaneous injection)	PEG-interferon alfa-2b	Merck & Co., Inc., Rahway, NJ, USA	Chronic Hepatitis C injection
Plegridy^®^ (peginterferon beta-1a) injection	PEG-interferon alfa-2b	Biogen, Cambridge, MA, USA	Multiple sclerosis
Spritam^®^ (levetiracetam) first 3D printed tablet	Levetiracetam	Aprecia Pharmaceuticals, Blue Ash, OH, USA	Myoclonic seizures

**Table 2:** Functionalized NGs-based approaches in cancer theranostics.

Functionalization/Coating	Nanogel Type and Composition	Encapsulation Moiety	Targeted Moiety/Cell Type	Applications	Reference
Folic acid	Amylopectin	Curcumin	Folate receptors	Colon cancer (HT29) therapy	[81]
Folic acid	PVP-co-acrylic acid	Doxorubicin, Bcl-2 siRNA	Folate receptors	FR targeting cancer therapy	[86]
Folic acid	Glycol chitosan	-	Folate receptors	FR targeted drug delivery (HeLa)	[87]
Folic acid	F/HA/Al/OXA	Oxaliplatin	CD44 receptor	antitumor on HT29 cell	[88]
Hyaluronic acid (HA)	Cholesteryl-HA	Etoposide, Salinomycin, Curcumin	CD44 receptor	Anticancer (MDR human breast and pancreatic adenocarcinoma)	[89]
Hyaluronic acid	Cholesteryl-HA	Curcumin	CD44 receptor	Anticancer (human pancreatic adenocarcinoma MIA PaCa-2)	[90]
Hyaluronic acid	Hyaluronic acid	Gold nanocluster, Doxorubicin	CD44 receptor	Anticancer and imaging	[91]
Chitosan	PEG-g-chitosan hydrogel	Cytotoxic T lymphocytes	Glioblastoma	Brain tumor immunotherapy	[92]
Dopa	PEG-based amphiphilic ABA-type triblock copolymer	Doxorubicin, ciprofloxacin	-	Drug delivery system for cancer and antimicrobial therapies	[93]
Tocopherol succinate (TOS)	HA-*ss*-TOS	Paclitaxel	CD44 receptor	Anticancer (Melanoma B16F10)	[94]
All-trans retinoid acid (ATRA) and TPENH_2_	HA-*ss*-ATRA/TPENH_2_	Doxorubicin	CD44 or LYCE-1 receptor	Antitumor and real-time intracellular imaging	[95]
C60 and TPENH_2_	HA-C60/TPENH_2_	Tirapazamine	CD44 receptor	Anticancer and imaging	[85]
Gd(III) chelates, folic acid	PEI-FA-PS	Copper (II) sulfide	Folate receptors	MR and PA imaging for PTT anticancer therapy	[83]
Galactose	CS-g-PNIPAm	Oridonin	Asialoglycoprotein-receptor	Liver cancer cells (HepG2)	[96]
Glucose/maltose	Poly(N-vinylcaprolactam)	-	Concanavalin A	Tumor immunotherapy and drug delivery	[97]
Doxorubicin or pDNA	CTCP	Doxorubicin or pDNA	-	Antitumor therapy	[98]
Doxorubicin	α-tocopherol polyethylene glycol 1000 succinate	-	-	Anticancer (MCF-7/ADR) therapy	[99]
AMD3100	Dextran	Doxorubicin	CXCR4	Antitumor and antimetastatic effect on breast cancer cells	[100]
Spermine	siRNA/CH-CA-Spe NG complex	siVEGF	Renal cell carcinoma	Intra-tumor gene delivery	[101]
Transferrin	PMEDAPA-based	Doxorubicin	Hepatoma (HepG2)	Chemotherapy and microwave heating cancer therapy on hepatoma	[102]
Diphtheria toxin receptor (DTR) ligand cross-reactive material 197 (CRM-197)	PEG-based	[^125^I]ITdU	Transcytosis via blood-brain carrier	Glioblastoma therapy	[103]
c(RGDFK) peptide	PBPLPs	Doxorubicin	α_v_β_3_ integrins of tumor vasculature	Targeted drug delivery, antitumor, and real-time fluorescence imaging PC3 (human prostate cancer)	[104]
Cyclo[Arg-Gly-Asp-D-Tyr-Lys]	poly(carboxybetaine methacrylate)	-	α_v_β_3_/α_v_β_5_ integrins	Targeted delivery to HUVECs for tumor angiogenesis inhibition	[105]
cRGDfC peptide, fluorescein	PEG-methacrylate based	-	avβ3 integrins of tumor vasculature	Targeted tumor imaging of MDA-MB-231 human breast cancer cells	[106]
RGD (Arg-Gly-Asp) peptide	Ethylene diamine and cholesteryl group-modified pullulan (CHP)	Protein (Drug Model)	αν integrin	Targeted drug delivery into HeLa cells	[107]
GE11 peptide and Hyaluronic acid	Sap-EGFR/CD44 HA	Saporin	Epithelial growth factor receptor and CD44 receptor	Inhibition of metastasis in breast cancer cells	[108]
Antisense oligonucleotides (ODN)	Poly(N-vinyl pyrrolidone)	-	-	Intracellular delivery of genetic materials	[109]
pDNA/siRNA	PGED	-	-	Anticancer (hepatoma) therapy	[110]
Anti-MUC1 aptamer	DNA tetrahedron	Doxorubicin, antisense oligonucleotide of *Bcl2* gene	Mucin 1 (MUC1)	Multidrug-resistant breast cancer therapy (MCF-7, MCF-7R) and gene therapy	[111]
MUC1 aptamer	PLA-PEG-Apt/Dox	Doxorubicin	Mucin 1 (MUC1)	Human alveolar basal epithelial cell (A-549)	[112]
Biotinylated α-CD3 and α-CD28 mAbs	Dendritic polyglycerol (dPG) and dPG-based	-	T lymphocytes	Activation of T cells	[113]
Cancer cell membrane (CCM)	DOX-loaded dual pH/oxidation-responsive NGs	DOX	Self-homing	Tumor-targeted chemotherapy	[114]

**Table 3:** Functionalized NGs-based approaches in vaccination.

Functionalization/Coating	Nanogel Type and Composition	Antigen	Targeted Moiety	Applications	Reference
Ovalbumin (OVA) and TLR7/8 agonist	P(mTEGMA)_25_-*b*-P(PFPMA)_34_ *	Ovalbumin (a model antigen)	-	Intravenous antitumor vaccination	[143]
All-trans retinal (adjuvant)	galactosyl dextran-retinal (GDR)/OVA	Ovalbumin (a model antigen)	-	Antitumor vaccination	[145]
Mannan	poly(2-hydroxyethyl methacrylate-*co*-methacrylic acid) P(HEMA-*co*-MAA)	Ovalbumin (a model antigen)	C-type lectin receptors (CLRs) on antigen-presenting cells	Delivery of encapsulated antigens for oral vaccination	[147]
Mannose	Pentafluorophenyl (PFP) activated ester	-	CD206 mannose receptor	Dendritic cell targeting (vaccine antigens and immunostimulatory molecules)	[148]
AT1R—PspA	cCHP	Angiotensin II type 1 receptor (AT1R), Pneumococcal surface protein A (PspA)	-	Intranasal vaccination against hypertension and pneumonia	[149]

* methoxy tri(ethylene glycol) methacrylate (mTEGMA) and pentafluorophenyl methacrylate (PFPMA).

**Table 4:** Functionalized NG-based approaches in wound healing, antiviral and antimicrobial therapy.

Functionalization/Coating	Nanogel Type and Composition	Encapsulation Moiety	Targeted Moiety	Applications	Reference
Sulfate (–OSO_3_^−^)	Dendritic polyglycerol sulfate	-	Viral glycoprotein	Antiviral (Herpes simplex virus type 1; HSV-1) inhibition	[155]
Sialic acid	Linear and Dendritic polyglycerol	-	Hemagglutinin protein	Influenza A viral inhibition to host cells (MDCK-II)	[159]
Lactosaminated-human serum albumin	LHSA-Mal-PLGA-Lam	Lamivudine	Asialoglycoprotein receptor (ASGP-R)	Hepatocyte-targeted antiretroviral drug delivery	[160]
Mannose	PEG/polyphosphoester (shell/core)	Vancomycin	Mannose receptor	Antibiotic delivery system to macrophages (antimicrobial)	[164]
Alcalase 2.4 L FG	Carbopol Aqua SF1	Ciprofloxacin	-	Antibiofilm on wound-associated bacteria (*S. aureus*, *P. aeruginosa*, *S. epidermidis*, *K. pneumoniae*, *E. coli*, *E. faecalis*)	[165]
Isoeugenol	Polyethylene glycol	-	-	Antibacterial against peri-implantitis bacteria (*Porphyromonas gingivalis*, *Prevotella intermedia*)	[168]
Poly(diallyldimethylammonium chloride)	Carbopol Aqua SF1	Chlorhexidine	-	Antibacterial, antifungal, and antialgal (*E. coli*, *S. aureus*, *S. cerevisiae*, *C. reinhardtii*)	[172]
Poly(diallyldimethylammonium chloride)	Polyacrylic acid	Berberine	-	Antibacterial (*E. coli*), antialgal (*C. reinhardtii*)	[173]
Polyethylenimine	Poly(maltose)	Sodium diclofenac	-	Antibacterial activity (*E. coli, S. aureus*)	[175]

**Table 5:** Functionalized NGs-based approaches in tissue engineering and regenerative medicine.

Functionalization/Coating	Nanogel Type and Composition	Encapsulation Moiety	Targeted Moiety/Cell Type	Applications	Reference
Cell integrin-binding peptide (YRGDS)	alpha-cyclodextrin nanobeads			Scaffold for tissue engineering and regenerative applications	[176]
BQ-123 and R-954	Chitosan and hyaluronic acid			Reduces inflammation and cartilage degradation in osteoarthritis	[178]
KAFAKLAARLYR (cell-penetrating peptide)	PNIPAM		Chondrocytes	Anti-inflammation of cartilage; intra-articular DDS	[179]
KAFAK (cell-penetrating peptide)	GC/Fu@KAFAK		Chondrocytes	Intra-articular injection of anti-inflammatory peptide-loaded NGs in osteoarthritis treatment	[180]
Laminin-111	Poly(ethylene glycol)	Nucleus pulposus cell		Cell delivery to intervertebral disk	[181]
NH2-Cy5	PEG-PEI	Rolipram	Activated Astrocytes	Targeted activation of astrocytes as neurodegenerative treatment	[182]
Polyethylenimine/VEGF_165_ pDNA	Heparin-Pluronic	Basic fibroblast growth factor	Ischemic limb (model system)	Endothelial cell differentiation and Neovascularization	[183]
Insulin	Polyvinylpyrrolidone	Insulin	Insulin receptors (IR) on neurons	Alzheimer’s disease therapy (neurodegenerative therapy)	[184]
Hyaluronic acid	Polyethylenimine	pDNA	CD44 receptor	Gene delivery in hMSCs	[185]

**Table 6:** Functionalized NGs-based approaches in other applications.

Functionalization/Coating	Nanogel Type and Composition	Targeted Moiety/Cell Type	Applications	Reference
Curosurf^®^ (Poractant alfa)	Dextran	Lung cell	Cytosolic siRNA gene delivery in lung epithelial cells (inhalation therapy)	[206]
Methyl sulfoxide side groups	Poly(pentafluorophyenyl methacrylate)	Viable epidermis	Topical transdermal delivery vehicle	[205]
4-methylumbelliferyl-β-D-glucuronide hydrate	Pullulan acetoacetate derivatives	β-glucuronidase-producing *E. coli*	Detection of bacterial enzymes/ bacteria	[207]
Hemoglobin	Dextran-*g*-succinic anhydride-*g*-dopamine	-	Oxygen carrier	[208]
Hemoglobin	HOOC-Dex-*g*-PNIPAAm	-	Oxygen carrier	[209]
Fe_3_O_4_ magnetic particles	GOX-hemin/polymer	-	Glucose detection	[210]
Ionic liquid	*Candida rugose* lipase-vinyl group	-	Biocatalysis in the synthesis of vitamin E succinate	[211]
Deferoxamine (DFO)	oxNG-DFO	Iron chelation	Iron chelation therapy	[212]
Aptamer	Polyacrylamide	-	Adenosine detection	[213]
Urokinase	PEG	thrombus	Thrombolytic treatment of ischemic stroke	[214]

## Data Availability

Not applicable.
